# Influence of Dietary Compounds on Arsenic Metabolism and Toxicity. Part I—Animal Model Studies

**DOI:** 10.3390/toxics9100258

**Published:** 2021-10-11

**Authors:** Monika Sijko, Lucyna Kozłowska

**Affiliations:** Department of Dietetics, Institute of Human Nutrition Sciences, Warsaw University of Life Sciences (SGGW-WULS), 159c Nowoursynowska Street, 02-776 Warsaw, Poland

**Keywords:** vitamins, minerals, inorganic arsenic, exposure, detoxification, metal toxicity, methylation

## Abstract

Population and laboratory studies indicate that exposure to various forms of arsenic (As) is associated with many adverse health effects; therefore, methods are being sought out to reduce them. Numerous studies focus on the effects of nutrients on inorganic As (iAs) metabolism and toxicity, mainly in animal models. Therefore, the aim of this review was to analyze the influence of methionine, betaine, choline, folic acid, vitamin B_2_, B_6_, B_12_ and zinc on the efficiency of iAs metabolism and the reduction of the severity of the whole spectrum of disorders related to iAs exposure. In this review, which includes 58 (in vivo and in vitro studies) original papers, we present the current knowledge in the area. In vitro and in vivo animal studies showed that methionine, choline, folic acid, vitamin B_2_, B_12_ and zinc reduced the adverse effects of exposure to iAs in the gastrointestinal, urinary, lymphatic, circulatory, nervous, and reproductive systems. On the other hand, it was observed that these compounds (methionine, choline, folic acid, vitamin B_2_, B_12_ and zinc) may increase iAs metabolism and reduce toxicity, whereas their deficiency or excess may impair iAs metabolism and increase iAs toxicity. Promising results of in vivo and in vitro on animal model studies show the possibility of using these nutrients in populations particularly exposed to As.

## 1. Introduction

**Figure 1:**
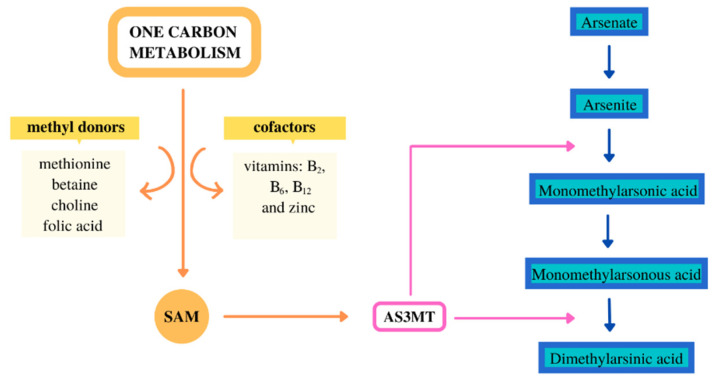
Arsenic metabolism and one carbon metabolism. AS3MT—arsenic (+3 oxidation state) methyltransferase; SAM—S-adenosylmethionine.

The metabolism of inorganic arsenic (iAs) was mainly analyzed in animal model studies, in vivo and in vitro. The iAs metabolism may vary considerably depending on the animal species [1]. Most mammals have the metabolism ability of iAs, and a limited metabolism ability has been observed in chimpanzees and marmoset monkeys [1,2]. The iAs metabolism in mice is much higher than in rats; this may be due to the fact that a large amount of DMA (dimethylarsinic acid) is accumulated in the red blood cells of rats [3]. The iAs metabolism involves alternate reactions of methylation and reduction to MMA (monomethylarsonic acid), DMA, and then these forms it are excreted by the kidneys. In most animal species, the major form of iAs excretion is DMA, and in humans, it can be excreted unchanged and as MMA and DMA [4,5,6]. The methylation reactions of iAs are catalyzed by an enzyme—arsenic (+3 oxidation state) methyltransferase [7]. S-adenosyl-methionine, which is the donor of methyl groups, is important in the methylation process. S-adenosyl-methionine is synthesized in the one-carbon-metabolism (OCM) pathway. Various dietary compounds are involved in the OCM, mainly as methyl group donors: methionine, choline, betaine, folic acid, and moreover, cofactors of the reaction—e.g., vitamin B_2_, B_6_, B_12_ and zinc (Figure 1) [8].

The many negative health effects caused by exposure to iAs lean, among others, to a deep analysis of the spectrum of health effects associated with As exposure and a search for ways that could reduce them on the effect of key dietary compounds on iAs metabolism and on reduction the adverse effects caused by iAs exposure. Therefore, the aim of this paper was to review and analyze the results of in vivo and in vitro studies on animal model on the influence of donors of methyl groups (methionine, choline, betaine, folic acid) and cofactors of reaction (vitamins B_2_, B_6_, B_12_ and zinc) on the efficiency of the metabolism process, as well as the reduction of the severity of the whole spectrum of disorders related to iAs exposure.

## 2. Methods

In this review, the electronic database PubMed was used. The following keywords were used to search for articles: arsenic and: methionine, betaine, choline, folic acid, folate, zinc, vitamin B, vitamin B_2_, vitamin B_6_, vitamin B_12_, riboflavin, pyridoxine, cobalamin. The review was based on: The PRISMA statement for reporting systematic reviews and meta-analyses of studies that evaluate health care interventions: explanation and elaboration [9]. The search results in 2434 articles, excluding those unrelated to the topic of the study and those that examined the effects of complex plant extracts. Overall, 58 (in vitro and in vivo studies on animal models) original peer-reviewed articles in English were included in the analysis, which studied the effects of: methionine, betaine, choline, folic acid, vitamin B_2_, B_6_, B_12_, zinc on iAs metabolism and As-induced toxicity. Articles published between 1980 and 2020 were used, of which 87.9% were published after 2000.

## 3. Results

### 3.1. Methionine

Six studies were conducted to examine the influence of methionine on iAs metabolism and decrease of iAs-induced toxicity using animal models. The experimental protocol included supplementation with different doses of methionine and administering a low-methionine diet with exposure to iAs for different time periods. Table 1 presents the results of these studies.

#### 3.1.1. Methionine—iAs Metabolism

The effects of supplementation and deficiency of methionine on iAs metabolism were evaluated by conducting five studies. In three studies, the metabolism iAs was altered by supplementation with methionine. The results showed a decrease in the levels of total arsenic (tAs), iAs, MMA in the blood, levels of tAs and %iAs in the liver, as well as levels of DMA and tAs in the brain. This study also revealed increased levels of DMA, primary methylation index in the blood, as well as enhanced levels of %DMA, primary methylation ratio, and secondary methylation index in the liver [10,11,12]. Two studies analyzed the influence of a low-methionine diet on iAs metabolism. This diet showed a negative effect which can be attributed to the disposition of iAs species in the tissues, resulting in increased concentration of iAs, %monomethyl-As, and pentavalent iAs, but decreased levels of dimethyl-As in the liver, and increased %iAs and decreased dimethyl-As in the kidney [13,14]. These studies also showed differences in the concentrations of As species in the urine sample. In the study by Vahter and Marafante [13], decreased levels of tAs, DMA and increased levels of iAs were observed. Furthermore, the urine sample of mice exposed to arsenic trioxide (accounting for clinical exposure) exhibited increased excretion of tAs and trivalent iAs species, but no statistically significant differences in arsenic excretion were observed in mice exposed to sodium arsenate (accounting for environmental exposure). These two groups of mice also revealed the increased expression of multidrug resistance-associated protein 1 (arsenic species transporter) [14].

#### 3.1.2. Methionine—Toxicity of iAs

The protective effect of methionine on the toxicity of iAs was analyzed in three studies. In a study using an animal model, exposure to iAs and supplementation with methionine showed hepatoprotective and renoprotective effects (decreased lipid peroxidation, and increased activity of antioxidant enzymes in kidney and liver) [12]. Methionine supplementation also had a beneficial effect on glucose homeostasis (restored normal blood glucose level, liver pyruvic acid level, free amino acid nitrogen concentration in the liver and kidney, glutamate-pyruvate transaminase activity in the kidney) in the group of rats fed with a methionine-rich diet and exposed to iAs [15].

In a study by Zhao et al. [11], it was shown that administration of methionine alleviated the negative effects of iAs exposure associated with elevated levels of nitric oxide in the brain.

#### 3.1.3. Methionine—Summary

In summary, the in vivo studies in an animal model exposed to iAs showed that methionine supplementation had hepatoprotective, renoprotective, neuroprotective, and antidiabetic effects, and potentially may also increase iAs metabolism. The beneficial effect was observed regardless of the method of administration, time of administration and dose of both methionine (intraperitoneally or orally for 5 days or 1, 4, 12 weeks; in different doses in range 25–400 mg/kg bw day and 0.8% of diet), as well as iAs (intraperitoneally or orally; for 21 days or 4, 12 weeks; in different doses: 50 mg/L, 10 ppm in drinking water, 5 mg/kg bw/day).

In turn, in two studies, the use of low-methionine diet for a long period of time (orally for 6 or 8 weeks) showed a negative effect on iAs metabolism (after just one dose of iAs—orally or intravenously in range 0.2–0.75 mg/kg bw).

### 3.2. Choline

The effect of choline on metabolism and on the reduction of iAs-induced toxicity was analyzed in four studies (two in vivo using animal models and two in vitro). The results are summarized in Table 2.

#### 3.2.1. Choline—iAs Metabolism

The association between choline-deficient diet and iAs metabolism was analyzed in two studies. Overall, treatment with a choline-deficient diet resulted in adverse effects, like decreased urinary excretion of tAs and DMA in the urine [13,16]. In addition, Vahter and Marafante [13] observed higher levels of MMA in urine, but increased tAs concentration in the liver, microsomes, and lungs.

#### 3.2.2. Choline—Toxicity of iAs

An in vitro study with guinea pig cardiomyocytes revealed the cardioprotective effect of choline (decreased QT prolongation, L-type calcium currents, and intracellular calcium concentration, which were enhanced by iAs) [17].

In chick embryos, neuroprotective effects of different doses of choline (25 µg/µL and 50 µg/µL) on halting the development of a neural tube defect were analyzed [18]. Only lower doses of choline were found to be effective in reducing the negative effects of iAs (increasing the survival rate and relative extra-embryonic vascular area, and reducing neural tube closure defects). In addition, it has been shown to alleviate the adverse effects of iAs exposure, such as the inhibition of differentiation of neural stem cells into neurons (which decreased the fluorescence signals of nanog and small C-terminal domain phosphatase 1, but increased the fluorescence signals of β3-Tubulin), decreased hypomethylation in the nervous system and spinal cord (which resulted, among other effects, in increased fluorescence intensity of anti-5-methylcytidine, and enhanced expression of DNA (deoxyribonucleic acid) methyltransferase 3A and DNA methyltransferase 1), and decreased apoptosis in chick embryos (which resulted in decreased expression of BCL2-associated X protein and increased expression of B-cell lymphoma protein 2 alpha).

Tice et al. [16] analyzed the influence of choline-deficient diet on iAs-induced toxicity. In the group of rats fed a choline-deficient diet and exposed to iAs (once or four times), DNA damage, particularly in skin cells (decreased DNA migration) and chromosomal damage in bone marrow (increased frequency of micronucleated polychromatic erythrocytes), was observed.

#### 3.2.3. Choline—Summary

Both in vitro and in vivo studies have shown that choline supplementation can reduce the adverse changes induced by iAs in the cardiovascular and nervous systems. In vivo and in vitro studies showed the cardioprotective effect, which was noticeable after a single dose of choline (8 mg/kg or 1 mM before, simultaneously with the exposure to iAs—1.6 mg/kg or 50 µM). In turn, neuroprotective effect was depended on the dose of choline (25 or 50 µg/µL), and was only observed at the lower dose.

Moreover, choline deficient diet (for a long period of time—2 or 6 weeks) and exposure to one dose of iAs (orally or intravenously in range 0.4–10 mg/kg) was found to increase DNA damage and decrease iAs excretion.

In these studies, not only deficiency, but also the dose of choline, appears to have a significant effect on the obtained results. High doses increased adverse effects.

### 3.3. Vitamin B_2_ with Selenium

Two studies evaluated the protective effect of riboflavin (and selenium nanoparticles) on reducing the adverse effects of iAs in *Pangasianodon hypophthalmus* reared in the presence of iAs and high temperature [19,20] (Table 3).

#### 3.3.1. Vitamin B_2_ with Selenium—iAs Metabolism

In the studies involving *Pangasianodon,* only tAs bioaccumulation in various organs was analyzed. Vitamin B_2_ supplementation caused a reduction in tAs concentration in the liver, gills, brain, kidneys, and muscles [19,20].

#### 3.3.2. Vitamin B_2_ with Selenium—Toxicity of iAs

Supplementation with these ingredients reduced oxidative stress, increased the levels of acetylcholine in the brain, and enhanced thermal tolerance, growth performance, and immunity [19,20].

#### 3.3.3. Vitamin B_2_ with Selenium—Summary

To sum up, the two in vivo studies show that vitamin B_2_ (with selenium) has a beneficial effect associated with the reduction of tAs bioaccumulation and with reduction of adverse iAs effect in digestive, nervous, respiratory, immune, and urinary systems. These two studies were carried out in the same animal model and used the same vitamin B_2_ and iAs doses, as well as exposure time. Three doses of vitamin B_2_ were used (5, 10, 15 mg/kg diet) and the beneficial effect was already demonstrated at the lowest dose. The favorable results could also be influenced by the long duration of the vitamin administration, the study lasted up to 96 days. It is not known whether supplementation with this vitamin would be equally effective in studies in other animal models and under different experimental protocols.

### 3.4. Vitamin B_12_

In the three studies performed using animal models exposed to iAs, protective effects of vitamin B_12_, such as enhanced metabolism and reduced toxicity, are analyzed (Table 4).

#### 3.4.1. Vitamin B_12_—iAs Metabolism

In one study, the association between vitamin B_12_ and iAs metabolism was examined. In this study conducted in a rat model, supplementation with vitamin B_12_ increased the excretion of tAs in the urine, which subsequently resulted in reduced tAs levels in the blood and liver [21].

#### 3.4.2. Vitamin B_12_—Toxicity of iAs

The protective action of vitamin B_12_ on the changes induced by iAs in the digestive system has been studied. In a study carried out in an animal model exposed to iAs, vitamin B_12_ was shown to have a hepatoprotective effect. In the liver of rats, increased activity of antioxidant markers, decreased activity of pro-oxidative markers and liver enzymes, beneficial effect on apoptotic changes, and decreased histopathological damage were observed [21]. In a rat model, Chen and Whanger [22] analyzed, inter alia, the relationship between vitamin B_12_ intake and levels of metallothionein. In the group that received vitamin B_12_ and was exposed to iAs, reduced levels of metallothionein in the liver were observed compared to the group that did not receive vitamin B_12_.

Acharyya et al. [23] showed that vitamin B_12_ supplementation caused a slight decrease in adverse effect of iAs not only in the digestive, but also urinary, and respiratory systems. The following changes were observed in the liver: decreased lipid peroxidation, decrease in the levels of liver function markers, DNA breakage, extensive damage to the histoarchitecture, and increased activity of antioxidants enzymes. In the kidney, improved levels of function marker and decreased tissue degeneration were noticed. In addition, decreased concentration of free radical products and increased activity of antioxidants were observed in the intestine and lungs.

#### 3.4.3. Vitamin B_12_—Summary

The aforementioned in vivo studies provide evidence for the beneficial effects of vitamin B_12_ supplementation on iAs metabolism and reduction of unfavorable changes in the digestive as well as, to a lesser extent, in the urinary and respiratory systems. The beneficial effect in the study on the rat model was observed regardless of the experimental protocol—the same method of vitamin B_12_ and iAs administration (orally), duration (for 28, 30 days or 8 weeks), but different doses of vitamin B_12_ administration (0.07 µg/100 g bw/day, 63 µg bw/day, 100 µg/kg diet) and iAs (3 mg/kg bw/day, 150 µg/g, 0.6 ppm/100 g bw/day).

### 3.5. Folic Acid

The influence of folic acid on metabolism and reduction of iAs-induced toxicity was investigated in 15 animal model studies and in 4 in vitro studies (Table 5).

#### 3.5.1. Folic Acid—iAs Metabolism

The studies performed on rats exposed to iAs proved the beneficial effect of folic acid supplementation, which was associated with increased tAs content in the urine samples and decreased levels in the blood, liver, and stool samples [21,24,25]. A similar effect was observed in a study conducted by Huang et al. [26] in mice (wild type and As3mt knockout) fed a low or high fat diet with supplementation with folic acid and exposed to iAs. This outcome was evident only in wild-type female mice exposed to iAs and higher dose of folic acid. A low-fat diet supplemented with folic acid increased iAs metabolism (decreased %iAs and increased %dimethyl As in urine), whereas a high-fat diet decreased its metabolism (decreased tAs, %iAs and increased %monomethyl and %dimethyl As in the liver). The group of mice (As3mt knockout) which have a limited metabolism capacity of iAs exhibited no significant differences in the levels of As species in the urine and liver. However, in another study, supplementation with folic acid increased iAs metabolism in the maternal livers, resulting in a reduced concentration of iAs and increased methylation ratios, but no influence on plasma concentrations of S-adenosylmethionine and S-adenosylhomocysteine was noticed. In the fetal livers, the beneficial effect of folic acid on iAs metabolism was not observed, and increased concentration of only S-adenosylhomocysteine was detected [27].

The adverse effects of folic acid deficiency were observed in two studies carried out in mice exposed to iAs. A folate-deficient diet decreased the urinary excretion of As in the wild-type mice (decreased %tAs in the urine) [28,29]. Moreover, folate-binding protein-1 (Folbp1) mice showed less efficient iAs metabolism—which was evidenced by the decreased percentage of arsenate and increased %DMA in the urine, but did not affect the plasma concentrations of S-adenosylmethionine and S-adenosylhomocysteine compared to wild-type mice [28]. However, no significant changes were observed in the group of Folbp2 mice, which showed no differences in the excretion of As, but revealed altered plasma concentrations of S-adenosylmethionine and S-adenosylhomocysteine [29]. 

#### 3.5.2. Folic Acid—Toxicity of iAs

The protective effect of folic acid following exposure to iAs has been analyzed with respect to a reduction in the damage caused to the digestive system. The two animal model studies demonstrated that folic acid exhibits hepatoprotective effect in rats exposed to iAs by decreasing the oxidative stress levels, lipid peroxidation, apoptosis, elevated serum levels of liver function markers, and tissue damage [21,23].

In the two studies conducted in rats exposed to iAs, the protective effect of folic acid was associated with a reduction in oxidative stress and lipid peroxidation in the pancreatic tissue [24,30]. One of the studies demonstrated a decrease in DNA damage, which was evident by reduced DNA smearing in pancreatic islet cell mitochondria and lymphocytes [24].

In one study, the supplementation of folic acid in rats exposed to iAs showed an adverse effect on gut flora, as it resulted in a decreased bacterial count in the stool [25]. In wild-type mice exposed to iAs and fed a high-fat diet with low folate dose, adverse effects on glucose homeostasis (marginal increase in fasting plasma insulin levels and homeostasis model assessment of insulin resistance) were observed [26].

A study by Tsang et al. [27] showed that supplementation with a high dose of folic acid may exert an adverse effect on the mouse fetal liver exposed to iAs, which is manifested through changes in DNA methylation and genes associated with cancer and development. 

Supplementation with folic acid alleviated the adverse effect of iAs in the urinary system by improving the reno-somatic index values and decreasing the biochemical marker of kidney function (serum urea level) in the rat kidney [23].

Three studies performed on animal models exposed to iAs demonstrated a cardioprotective effect of folic acid. Folate-sufficient diet decreased genotoxicity in mice exposed to iAs (decreased chromosomal damage by reducing the incidence of micronuclei formation in polychromatic erythrocytes and normochromatic erythrocytes) in comparison to mice fed with a folate-deficient diet and exposed to iAs [31]. Two studies conducted in female rats exposed to iAs showed that folic acid supplementation exerted a protective effect on embryonic cardiac defects. This outcome was more prominent when supplemented with higher doses of folic acid (5.3 and 10.6 mg/kg bw/day) [32,33]. Supplementation with this vitamin has been shown to increase embryonic growth and development [32,33], via upregulating the gene expression of cardiac transcription factors [32], downregulating the expression of genes involved in cardiac development, and decreasing the incidence of protein acetylation and cardiac malformation [33].

The influence of folic acid on the changes induced by iAs in the nervous system has also been studied. Supplementation with folic acid in three mice strains exposed to iAs exhibited no protective effect on reducing the frequency of neural tube defects in embryos but rather was found to increase embryo/fetal lethality; on the other hand, folate supplementation caused increased maternal lethality [34]. The study on female mice with genotypes Folbp2^+^/^+^ and Folbp2^−^/^−^ showed an increase in the incidence of exencephaly, and elevated growth was noticeable in Folbp2^−^/^−^ mice and in the group of mice fed with a folate-deficient diet [35]. In an in vitro study performed on zebrafish embryos, folic acid showed a protective effect against adverse effects of iAs. In the embryo, a beneficial effect was associated with increased survival and maintenance of normal development, via decreasing the defects in cardiac and nervous systems and upregulating the expression of decapentaplegic and Vg-related-1 protein [36]. In addition, folic acid deficiency caused an increase in the iAs-induced neurotoxicity in NB2a/dl cells by inducing changes in the neurofilament dynamics, thus resulting in decreased neurofilament transport and increased immunoreactivity of perikaryal RT97 and perikaryal phospho-NF [37].

The protective action of folic acid on the adverse effects of iAs was also analyzed in skin cells. In the one study on mice exposed to iAs, a folic acid-deficient diet negatively affected the skin cell proliferation and differentiation by decreasing the expression of key genes involved in this process (including those involved in epidermal development and differentiation) and increasing the expression of cancer-related genes (cellular movement genes) [38].

A reduction in iAs-induced toxicity was observed in SWV/Fnn embryo fibroblasts following folic acid treatment, but the effect was found to be dose-dependent. A significant reduction in cytotoxicity, resulting in the increased viability of cells, was noted at a folic acid concentration of 270 µM in the media, containing a constant concentration of As or DMA. Furthermore, when cells were treated with higher doses of iAs or DMA, folic acid supplementation did not affect the viability of cells. Moreover, the treatment of cells with iAs and 90 µM concentration of folic acid caused an increase in the number of cells in the media at 7 days, but not at 6 days of treatment [39]. Exposure of Folbp2 null fibroblasts to iAs and supplementation with folic acid did not show any protective effect on survival, but exposure to this element certainly decreased the uptake of folic acid. In contrast, exposure of Folbp2 wild-type fibroblasts to folic acid increased the survival rate, but did not affect the folic acid uptake [40].

#### 3.5.3. Folic Acid—Summary

The studies performed in vivo on animal models exposed to iAs have shown not only alleviating effects of folic acid on digestive, urinary, and circulatory systems, but also potentially can increase iAs metabolism. These beneficial effects were observed during oral administration with different time and doses of folic acid (for 28, 30 days; 6, 7 weeks; 5.3, 10.6 or 36 µg/kg bw/day; 5 or 10 mg/kg diet; 4 µg/100g bw/day), as well as iAs (for 4, 28, 30 days; 6 weeks; 2.5, 3, 5, 10 mg/kg bw/day; 100 ppb in drinking water; 0.6 ppb/100 g bw/day; 75 mg/L).

However, the negative effects were also observed. In the four in vivo studies, the supplementation of folic acid had an adverse effect on metabolism iAs, gut flora, DNA methylation, neural development and viability. Various modes of administration, exposure time, and dose of folic acid (orally or intraperitoneally; for 2, 13 days or 2, 8 weeks; 200 µg/day; 10, 11 mg/kg/diet; 2 or 25 mg/kg bw/day) and iAs (orally or intraperitoneally; for 2, 10 days; 2, 13 weeks; 1 mg/L; 85 ppm in drinking water; 40 mg/kg bw/day; 100 ppb in drinking water) were used in these studies. In the case of an unfavorable effect of supplementation with folic acid on iAs metabolism, the effect may result from the different diets (the combination of a high-fat diet and folic acid supplementation decreased the iAs metabolism, as opposed to a low-fat diet). The results were also influenced by species differences and different models of animals (wild-type mice and mice with limited capacity to methylation), as well as the stage of development, were also important (beneficial effect in maternal, but not in the fetal). In the remaining studies, an adverse effect may result from the use of high doses of folic acid. 

The five studies carried out in mice exposure to iAs and fed with a folate-deficient diet showed adverse effects related to iAs metabolism, glucose homeostasis, development, and skin proliferation. In three studies, iAs was administered intraperitoneally (a single dose—10 µL/g bw; twice at dose—40 mg/kg), and in the two other studies, it was administered orally for 30 days or 13 weeks at a dose of 1 ppm; 100 ppb in drinking water. Despite differences in study protocols, folic acid deficiency exacerbated the adverse effects of iAs. The results could also be influenced by animal species differences (the studies were carried out in wild-type and mice Folbp1^−^/^−^ and 2^−^/^−^ mice), as well as by diet (a high-fat diet had adverse effects).

In the in vitro studies (iAs exposure ranged from 0.3–100 μM and 2–10 mM of iAs; duration of exposure 24 h), folic acid showed cardioprotective, neuroprotective and anti-cytotoxic effects. One study showed a beneficial effect with two (50 and 100 µM) doses of folic acid, with the higher dose showing a more pronounced beneficial effect. In the second of these studies, the constant concentration of folic acid brought the expected positive results, and in the third study, the beneficial effect may be due to the length of treatment with folic acid (up to 1 week). In one in vitro study, folic acid, did not increase survival, but the result was influenced by the type of cells used (cells lacking Folbp 2). In one in vitro study, it was found that folate deficiency enhanced the neurotoxicity of iAs at dose 0.07 µm for 24 h.

### 3.6. Vitamin B_12_ and Folic Acid

Five studies have been conducted to study the modulating effect of simultaneous supplementation with vitamin B_12_ and folic acid on iAs metabolism and toxicity in animal models (Table 6).

#### 3.6.1. Vitamin B_12_ and Folic Acid—iAs Metabolism

The study performed on rats exposed to iAs demonstrated that the beneficial effect of supplementation with vitamin B_12_ and folic acid was associated with the more efficient excretion of tAs, which was evident by increased concentration of tAs in urine and decreased levels in blood and liver [21,24]. However, in another study, no significant changes in tAs excretion were observed in the female adult mice that were supplemented with these vitamins. In the group which received supplementation and was exposed to low doses of iAs, only an increase in the concentrations of monomethylarsenic and dimethylarsenic in the urine was shown [41].

#### 3.6.2. Vitamin B_12_ and Folic Acid—Toxicity of iAs

Five studies using animal models aimed to determine the influence of the simultaneous application of vitamin B_12_ and folic acid on the adverse effects of iAs in the digestive system. These vitamins demonstrated hepatoprotective effects in mice, which were associated with a reduction in oxidative stress levels, lipid peroxidation, DNA fragmentation, damage to the histoarchitecture of the liver, as well as restored the serum level of liver function markers [21,42]. Moreover, they improved the lipid profile [42] and decreased hepatic mitochondrial apoptotic changes [21].

Two studies analyzed the protective effect of vitamin B_12_ and folic acid in reducing the damage to pancreatic islet cells of rats exposed to iAs. Supplementation with these vitamins decreased the production of reactive oxidants, reduced lipid peroxidation, increased the activity of antioxidative enzymes [24,30], decreased DNA damage [24], decreased the levels of inflammatory markers, and increased islet cell counts [30].

In another study, the protective effect of simultaneous application of vitamin B_12_ and folic acid on disorders associated with glucose metabolism was investigated in the offspring of mice prenatally exposed to iAs. Glucose metabolism disorders were mainly reported in male offspring, but prenatal vitamin B_12_ and folic acid supplementation reduced hyperglycemia and insulin resistance in the mice exposed to lower and higher doses of iAs, resulting in a marginal decrease in the fasting plasma insulin levels and Homeostasis Model Assessment of Insulin Resistance. Moreover, in the group including male offspring mice exposed to 1000 ppm of iAs, supplementation with these vitamins increased global DNA methylation in the liver [41].

The study by Acharyya et al. [23] investigated the influence of cotreatment of vitamin B_12_ with folic acid on the adverse effect of iAs, and found to be highly effective in restoring the damage to the digestive system. The vitamins restored normal levels of liver and renal function markers, decreased oxidative stress and lipid peroxidation in the liver, intestine, kidney, and lungs, as well as repaired DNA damage in the liver and kidney. Additionally, supplementation with these vitamins prevented hepatic and renal tissue degeneration.

#### 3.6.3. Vitamin B_12_ and Folic Acid—Summary

The in vivo studies in animal models revealed that simultaneous supplementation with vitamin B_12_ and folic acid exerted a positive effect on iAs metabolism and alleviated adverse effects, not only in the digestive system, but also in the urinary and respiratory systems. In one of these studies, the supplementation of these vitamins had a positive effect on iAs metabolism only at a lower exposure dose, which may indicate that the beneficial properties of these vitamins did not exceed the negative effects of a higher dose of iAs, or showed a synergistic effect with this element. In the remaining studies on rat models, different doses of vitamins (vitamin B_12_: 0.63 µg/kg bw/day; 0.07 µg/0.1 mL water/100 g bw/day; 0.07 µg/100 g bw/day and folic acid: 3 mg/kg bw/day; 36 µg/kg bw/day; 4 µg/100 g bw/day; 4 µg/0.1 mL water/100 g bw) and iAs (3 mg/kg bw/day; 0.4 or 0.6 ppm/100 g bw/day; 100, 1000 ppb in drinking water) and similar exposure times (24, 28, 30 days) were used, however, using each of these protocols, beneficial effects in reducing the adverse effects of iAs were obtained.

### 3.7. Zinc

The protective effect of zinc has been analyzed by performing studies in 21 animal models and 6 cell lines that were exposed to iAs. The results of these experiments are summarized in Table 7.

#### 3.7.1. Zinc—iAs Metabolism

Zinc supplementation in the animal models exposed to iAs decreased tAs levels in liver [43], kidney [44], spleen [45], and brain [46,47], but it had no effect on tAs concentration in the blood, liver, and kidney in two studies [48,49]. Pretreatment with zinc enhanced the elimination of As, which was evidenced by a reduced concentration of As-73 in the blood, skin, muscle, and organs such as heart, lung, kidney, and small intestine, but similar effects were not observed in the liver, brain, and large intestine [50]. In one in vitro study conducted in SA7 cells exposed to iAs, zinc pretreatment resulted in decreased tAs accumulation and increased excretion [51].

#### 3.7.2. Zinc—Toxicity of iAs

Many studies analyzed the protective effect of zinc on the digestive system. In a study conducted in common carp exposed to iAs, the anterior and mid-intestines showed increased activity of superoxide dismutase and tight junction proteins (inter alia, mRNA levels of occludin, claudin, and zonula occludens), as well as decreased levels of inflammatory markers (mRNA levels of interleukins [1β, 6], phosphorylation of inhibitor of nuclear factor kappa B, and nuclear factor kappa B nuclear translocation) and histological changes in intestines [52].

In three studies, zinc has been shown to exhibit a hepatoprotective effect, which is accompanied by a reduction in oxidative stress [48,53,54], lipid peroxidation [48,54], apoptosis [53], and damage of the liver structure [53,54], as well as with the elevation of blood levels of alanine and aspartate transaminases [48], metallothionein expression [54], and activity of δ-aminolevulinic acid dehydratase [48].

In one study, zinc supplementation after exposure of male mice to iAs did not reduce the adverse effects (oxidative stress, lipid peroxidation in the liver), while simultaneous administration showed a partial mitigating effect, mediated by increased δ-aminolevulinic acid dehydratase activity in blood, and decreased lipid peroxidation and oxidized glutathione in liver [49].

Zinc supplementation in the group of rats exposed to iAs showed an attenuating effect in regulating the biokinetics of ^65^Zn, which was impaired in the group of rats exposed to iAs, but not treated with zinc. It was observed that zinc decreased the fast component in the liver and decreased the uptake of ^65^Zn in the brain and liver [43].

Furthermore, the in vivo (rat liver) study carried out in the presence of iAs demonstrated that zinc deficiency increased inflammatory response (inter alia through increased production of inflammatory markers) [55].

An in vitro study showed that zinc deficiency and iAs exposure adversely affected pancreatic beta cells. In cells, deficiency resulted in, inter alia, increased apoptosis (increased poly(ADP) polymerase), DNA damage (increased the levels of a marker of DNA breaks), and decreased proliferation (decreased viable cells) were observed [56].

Six studies analyzed the potential role of zinc in the reduction of iAs-induced toxicity in the urinary system. The renoprotective effect of zinc was observed in three studies carried out in animal models exposed to iAs [44,57,58]. The protective effect of zinc on the kidneys was not only by reducing oxidative stress, but also by reducing tight junction damage, mitigating disturbances in protein homeostasis, and reducing autophagy [44]. In the second study, zinc also reduced oxidative stress, lipid peroxidation, protein and DNA damage, apoptosis, inflammation, and kidney histopathological changes [57]. Supplementation with zinc during gestation and lactation in female rats also reduced the adverse effect of iAs in the offspring (by reducing lipid peroxidation and changes in the structure of the kidney) [58]. In two studies, zinc did not exhibit renoprotective effects [48,49].

In turn, zinc deficiency in chickens exposed to iAs increased plasma levels of uric acid and urea and enhanced arginase activity in the kidney [59].

Zinc showed a protective effect on the lymphatic system of carps exposed to iAs by decreasing the toxicity-related changes in the spleen, inter alia, by decreasing harmful changes in spleen tissue, the expression of genes related to endoplasmic reticulum stress (glucose-related protein 78 and 94, PKR-like reticulum kinase, C/EBP homologous protein), apoptosis (apoptosis antigen 1, caspases 3, 8, 9, Bcl-2-associated X protein), and autophagy (Beclin-1, autophagy-related 5, microtubule-associated protein 1 light chain 3) [45].

The protective effect of zinc on the circulatory system has also been demonstrated. The heart tissue of common carp exposed to iAs revealed reduced oxidative stress (through decreased production of reactive oxygen species and increased activity of antioxidant enzymes), lipid peroxidation (through decreased content of malondialdehyde), apoptosis (through increased expression of Bcl-2 and decreased expression of Bax and caspases), autophagy (by decreasing the level of proteins involved in the previously mentioned pathways), and injury symptoms [60]. In rats exposed to iAs, zinc supplementation reduced the damage to erythrocytes, mediated through the increased activity of antioxidant enzymes, decreased lipid peroxidation in the serum, and decreased morphological changes in red blood cells [61].

Three studies conducted in animal models exposed to iAs have also shown that zinc has a protective effect on the nervous system. Zinc decreased neurotoxicity in the rats belonging to three age groups (young, adult, and old) by lowering behavioral perturbations and alleviating perturbations in the cholinergic system (through increased activity of acetylcholine and decreased amount of acetylcholine in the brain) [46]. The protective effect of zinc was also observed in a study performed in groups of rats belonging to different age groups (21 and 28 postnatal days and 3 months old). In all age groups, zinc reduced oxidative stress (by increasing the activity of antioxidant enzymes), decreased lipid peroxidation (by reducing malondialdehyde concentration), and also decreased apoptosis (by decreasing mRNA expression of caspase) [47]. In another study, zinc also reduced adverse effect of iAs in the offspring mice (through increased morphological development, decreased early development of sensory–motor reflexes, increased motor behavior, and decreased oxidative stress in the serum) [62]. In an in vitro study, higher doses of zinc (50 and 75 µM) reduced apoptosis in a neuronal cell line (by reducing DEVD-caspase activity), but such an effect was not observed at lower doses of Zn (25 µM) [63].

A study carried out by Kreppel et al. [50] in an animal model revealed that zinc pretreatment reduced As-induced lethality, but no significant correlation between metalothionein induction in the liver and protection against the lethal effect of As by zinc was noted. In another study, both pretreatment with zinc and simultaneous administration did not reduce teratogenicity in mice and embryos exposed to iAs, which was evident by no significant changes in maternal, placental, and fetal weight, and no reduction of malformation in the fetuses and morphological development in the embryo [64].

Zinc deficiency in the embryos of zebrafish exposed to iAs did not affect mortality and development, but an adverse effect was observed with regard to the reduction of activity of the embryos and genes associated with oxidative stress and insulin production (decreased mRNA levels of 8-oxoguanine DNA glycosylase, nuclear factor (erythroid-derived 2)-like 2, and paired box 4) [65]. In the chickens that were fed a zinc-deficient diet and exposed to iAs, slower growth and increased hematocrit and activity of plasma alkaline phosphatase in the plasma were observed [66]. 

One of the studies showed that zinc reduced damage to the reproductive system of rats exposed to iAs, mainly through increasing the proportion of normal sperm and decreasing the abnormalities in spermatozoa [67].

#### 3.7.3. Zinc—Summary

In vivo studies have been shown that zinc reduced adverse changes induced by iAs in many systems, including digestive, urinary, lymphatic, cardiovascular, nervous, and reproductive, and can reduce the bioaccumulation of tAs in the many organs. The same methods of administration and exposure time (orally or subcutaneously; one dose or for 5, 15, 42, 60 days; 1, 3, 12 weeks; 1, 3 months), but different doses of zinc (1, 227 mg/L; 0.02% or 10 ppm in drinking water; 5, 10, 20 mg/kg bw/day; 153, 1000 µmol/kg bw) and iAs (10, 100 ppm in drinking water; 2.83, 100 mg/L; 2, 5, 10, 40 mg/kg bw/day; 75, 85, 115 µmol/kg bw), were used in the experimental protocols, although beneficial effects were obtained.

However, in four in vivo studies, zinc did not decrease: oxidative stress, teratogencity and tAs accumulation in the blood, liver, kidney, brain, large intestine. In these studies, the ingredients were administered for different periods of time and doses—zinc (for 1, 2, 5 days or 3 weeks; 5, 10, 20, 40 mg/kg bw/day) and iAs (for 1, 5 days; 3 weeks; 2, 45 mg/kg bw/day). The reason for the lack of beneficial effects in these 4 studies in comparison to the studies where the zinc effect was satisfactory may be the method of iAs administration. When iAs was administered orally for 3 weeks (1 study), a beneficial effect was seen only in the liver, but not in the kidneys (which may indicate differences in iAs metabolism in these organs). In the other studies, where iAs was administered intraperitoneally and subcutaneously (only one dose or one dose per day for 5 days), no beneficial effect of zinc was observed. Perhaps when iAs was taken orally, its absorption was limited, and therefore, in the case of direct administration (intraperitoneally and subcutaneously), the oral administration of zinc cannot reduce its negative effects.

In the four in vivo studies, zinc deficiency intensified the adverse effect of exposure to iAs (inflammation in the liver, disturbance in the urinary system, decreased growth and influence on expression of genes responsible to oxidative stress and insulin production). These adverse effects were observed in different experimental protocols. In three studies, zinc and iAs were administered orally, the time of exposure was the same (for 28, 32 days; 6, 8 weeks), but doses of zinc (6 mg/kg/diet; 2.5, 5, 14.45 µg/g/diet,) and iAs (50, 500 ppb in drinking water; 2µg/g) were different. The results of these studies may have been influenced by long exposure to iAs and a long period of zinc deficiency. Moreover, in one of these in vivo studies, the protocol of the experiment could have had a major impact on the results, zinc deficiency was applied to parental fish, and then embryos were exposed to iAs.

Furthermore, in two in vitro studies, zinc increased the excretion of tAs, as well as showed antiapoptotic effects. In one of these studies, the time of administration could be crucial—zinc was given before exposure to iAs. In the second study, the zinc dose appeared to be the outcome determinant. Antiapoptotic effects were shown with higher doses (50 µM or 70 µM), but a lower dose (25 µM) did not have much of an effect.

In one in vitro study after 5 days of zinc deficiency and after 24 h of exposure to iAs, intensified apoptosis and DNA damage, as well as decreased proliferation, were observed.

## 4. Conclusions

The results of the in vitro and in vivo animal model studies indicate that dietary compounds involved in iAs metabolism may have beneficial effects in reducing the severity of the entire spectrum of disorders associated with exposure to iAs. Numerous studies where the effects of folic acid and zinc have been analyzed allow one to draw some conclusions in terms of the role of these nutrients in iAs metabolism and the adverse effect reduction. Folic acid and zinc supplementation improved iAs metabolism and reduced adverse changes induced by iAs in many systems: digestive, urinary, cardiovascular, lymphatic, nervous and reproductive. Adverse effects of folic acid supplementation were also observed, and were mainly connected with reduction iAs metabolism, intensification of oxidative stress, and disturbances in: DNA methylation, gut flora composition, neural development, and viability. These adverse effects were determined by such factors as: type of diet (high-fat diet), type of animal model (mice with limited capacity to methylation), animal species (wild-type mice) and folic acid dose (high dose). Moreover, folate and zinc deficiency intensified the adverse effect of iAs exposure. The folate-deficient diet induced adverse effects related to iAs methylation, glucose homeostasis, development, and skin proliferation. Meanwhile, zinc deficiency intensified such adverse effects as: inflammation in the liver, disturbance in the urinary system, decreased growth, expression of genes responsible to oxidative stress and insulin production. The amount of research on the role of methionine, choline, vitamin B_2_, B_12_, and a combination of vitamin B_12_ and folic acid, zinc is very limited, and therefore, no meaningful conclusions can be drawn. Nevertheless, these few studies provide evidence for beneficial effects of methionine, choline, vitamin B_2_, B_12_, combination of vitamin B_12_ and folic acid on iAs metabolism and reduction unfavorable changes in digestive, urinary, nervous, cardiovascular, respiratory, immune systems. Among these ingredients, it has been shown that higher doses of choline are not effective in the reduction of adverse effects of iAs, and the deficiency of methionine and choline may impair iAs metabolism and contribute to DNA damages. In the case of these compounds, further studies are needed to fully determine their role in terms of iAs metabolism and reduction of the adverse health effects. Nevertheless, taking into consideration the promising results of in vivo and in vitro animal model studies, it seems reasonable to analyze the effect of these dietary components in populations exposed to As.

## Figures and Tables

**Table 1:** Results of in vivo studies with iAs exposure and methionine treatment.

Reference	Research Model	Study Description	Main Results
Jin et al., 2010 [10]	Mice, Albino, adult, female	CG (*n* = 8)—sodium arsenite through drinking water 50 mg/L (orally, for 5 weeks) G1 (*n* = 8)—sodium arsenite through drinking water 50 mg/L (orally, for 4 weeks) and after that methionine 200 mg/kg bw/day (IP injection, for 7 days) and sodium arsenite through drinking water 50 mg/L (orally, for 7 days)	G1 vs. CG blood: DMA↑, %iAs↓, PMI↑ liver: SMI↑
Zhao et al., 2011 [11]	Mice, Albino, adult, female	CG (*n* = 8)—sodium arsenite through drinking water 50 mg/L (orally, for 4 weeks) G1 (*n* = 8)—methionine 100 mg/kg bw/day (IP injection, for 4 weeks) and sodium arsenite through drinking water 50 mg/L (orally, for 4 weeks) G2 (*n* = 8)—methionine 200 mg/kg bw/day (IP injection, for 4 weeks) and sodium arsenite through drinking water 50 mg/L (orally, for 4 weeks) G3 (*n* = 8)—methionine 400 mg/kg bw/day (IP injection, for 4 weeks) and sodium arsenite through drinking water 50 mg/L (orally, for 4 weeks)	G1, G2, G3 vs. CG blood: iAs↓, MMA↓, tAs↓ brain: DMA↓, tAs↓
			G2, G3 vs. CG liver: DMA↑, %iAs↓, %DMA↑, %PMR↑
			G3 vs. CG brain NO↑
Nandi et al., 2005 [12]	Rats, Wistar albino, adult, male	CG (*n* = 6)—sodium m-arsenite (III) 10 ppm in drinking water (orally, for 12 weeks) G1 (*n* = 6)—sodium m-arsenite (III) 10 ppm in drinking water (orally, for 12 weeks) and methionine solved in distilled water 25 mg/kg bw/day (orally, for 12 weeks)	G1 vs. GC blood: tAs↓ erythrocytes: LPO↔, SOD↔, CAT↔ liver: tAs↓, LPO↓, SOD↔, CAT↑ kidney: tAs↓, LPO↓, SOD↔, CAT↑
Vahter and Marafante 1987 [13]	Rabbits, Swedish loop, adult, male	CG (*n* = 4)—standard diet (orally, for 6 weeks) and after that [^76^As] arsenite 0.4 mg/kg bw (IV injection, single dose) and sacrificed after 72 h G1 (*n* = 4)—low methionine diet 1.3 mg/kg (orally, for 6 weeks) and after that [^76^As] arsenite 0.4 mg/kg bw (IV injection, single dose) and sacrificed after 72 h	G1 vs. CG liver and microsomes: ^76^As↑ urine: tAs↓, iAs↑, DMA↓
Canet et al., 2012 [14]	Mice, C57BL/6, adult, male	CG (*n* = 3)—control diet (orally, for 8 weeks) and after that arsenic trioxide 0.2 mg/kg (orally, single dose) and sacrificed after 24 h G1 (*n* = 5)—methionine-and choline deficient diet (orally, for 8 weeks) and after that arsenic trioxide 0.2 mg/kg (orally, single dose) and sacrificed after 24 h	G1 vs. CG liver: %MMA↑, %DMA↓, %pentavalent iAs↑, expression of Mrp1 protein↑ kidney: % iAs↑, %DMA↓ urine: %tAs↑, %iAs↓, %trivalent iAs↑
		CG (*n* = 3)—control diet (orally, for 8 weeks) and after that sodium arsenate 0.75 mg/kg (orally, single dose) and sacrificed after 24 h G1 (*n* = 5)—methionine-choline deficient diet (orally, for 8 weeks) and after that sodium arsenate 0.75 mg/kg (orally, single dose) and sacrificed after 24 h	G2 vs. CG liver: %MMA↑, %DMA↓, %pentavalent iAs↑, expression of Mrp1 protein↑ kidney: %iAs↑, %DMA↓ urine: %tAs↔, %iAs↔, %trivalent iAs↔
Pal and Chatterjee 2004 [15]	Rats, Wistar, adult, male	CG (*n* = 6)—sodium arsenite 5.55 mg/kg bw/day (IP injection, for 21 days) G1 (*n* = 6)—sodium arsenite 5.55 mg/kg bw/day (IP injection, for 21 days) and after that 18% protein diet supplemented with 0.8% methionine (orally, for 5 days prior to sacrifice)	G1 vs. CG blood: glucose↑ liver: free amino acid nitrogen↓, pyruvic acid↑ kidney: free amino acid nitrogen↑, GPT↑

↑—significant increase; ↓—significant decrease; ↔—no significant changes; ALT—alanine aminotransferase; CAT—catalase; CG—control group; DMA—dimethylarsinic acid; G1—Group 1; G2—group 2; G3—group 3; GPT—glutamate-pyruvate transaminase; iAs—inorganic arsenic; IP—intraperitoneally; IV–intravenous; LPO—lipid peroxidation; MMA—monomethylarsonic acid; Mrp1—multidrug resistance-associated protein 1; NO—nitric oxide; PMI—primary methylation index; PMR—primary methylation ratio; SMI—secondary methylation index; SOD—superoxide dismutase; tAs—total arsenic species.

**Table 2:** Results of in vivo and in vitro studies with iAs exposure and choline treatment.

Reference	Research Model	Study Description	Main Results
Vahter and Marafante 1987 [13]	Rabbits, Swedish loop, adult, male	CG (*n* = 4)—standard diet (orally, for 6 weeks) and after that [^76^As] arsenite 0.4 mg/kg bw (IV injection, single dose) and sacrificed after 72 h G1 (*n* = 4)—choline deprived diet 1.3 mg/kg (orally, for 6 weeks) and after that [^76^As] arsenite 0.4 mg/kg bw (IV injection, single dose) and sacrificed after 72 h	G1 vs. CG liver, lung, microsomes: ^76^As↑ urine: tAs↓, iAs↔, MMA↑, DMA↓
Tice et al., 1997 [16]	Mice, B6C3Fl, adult, male	G1 (*n* = 4)—choline-sufficient diet (orally, for 1 week) after that choline-sufficient diet (orally, for 2 weeks) and sodium arsenite 2.5 or 5 or 10 mg/kg (orally, single dose) G2 (*n* = 4)—choline-sufficient diet (orally, for 1 week) and after that choline-deficient diet (orally, for 2 weeks) and sodium arsenite 2.5 or 5 or 10 mg/kg (orally, single dose)	in G1 bone marrow: MN-PCE↔, %PCE↔ liver parenchymal cells: DNA migration↓ bladder cells: DNA migration↓ lung cells: DNA migration↔ skin cells: DNA migration↔ bone marrow: MN-PCE↔, %PCE↓
			in G2 urine: tAs↓, DMA↓ liver parenchymal cells: DNA migration↔ bladder cells: DNA migration↔ lung cells: DNA migration↔ skin cells: DNA migration↓ bone marrow: MN-PCE↔, %PCE↔
		G1 (*n* = 4)—choline-sufficient diet (orally, for 1 week) after that choline-sufficient diet (orally, for 2 weeks) and sodium arsenite 2.5 or 5 or 10 mg/kg/day (orally, for 4 days) G2 (*n* = 4)—choline-sufficient diet (orally, for 1 week) and after that choline-deficient diet (orally, for 2 weeks) and sodium arsenite 2.5 or 5 or 10 mg/kg/day (orally, for 4 days)	in G1 liver parenchymal cells: DNA migration↓ bladder cells: DNA migration↓ lung cells: DNA migration↔ skin cells: DNA migration↔ bone marrow: MN-PCE↑, %PCE↓
			in G2 liver parenchymal cells: DNA migration↔ bladder cells: DNA migration↔ lung cells: DNA migration↔ skin cells: DNA migration↓ bone marrow: MN-PCE↑, PCE↔
Sun et al., 2006 [17]	cardiomiocyte (Guinea Pig, adult, female and male)	CG (*n* = 8)—arsenic trioxide 1.6 mg/kg (IV injection, single dose) G1 (*n* = 7)—choline 8 mg/kg (single dose) and after that arsenic trioxide 1.6 mg/kg (IV injection, single dose)	G1 vs. CG in vivo: after 120 min—QTc prolongation↓
		CG (*n* = 8)—arsenic trioxide 50 µM (single dose) G1 (*n* = 8)—choline 1 mM (single dose) and after that arsenic trioxide 50 µM (single dose)	G1 vs. CG in vitro: APD prolongation↓, I_Ca-L_↓
		CG (*n* = 8)—arsenic trioxide 50 µM (single dose) G1 (*n* = 8)—choline 1 mM (single dose) and arsenic trioxide 50 µM (single dose) and KCl 60 mM (single dose)	G1 vs. CG in vitro: changes of [Ca^2+^]_i_↓
Song et al., 2012 [18]	Chick embryos, White Leghorn	CG (*n* = 8)—sodium arsenite 100 nM (injected into the center of the egg yolk, incubation for 3 days) G1 (*n* = 6)—choline 25 µg/µL (injected into the center of the egg yolk, incubation for 3 days) and sodium arsenite 100 nM (injected into the center of the egg yolk, incubation for 3 days) G2 (*n* = 6)—choline 50 µg/µL (injected into the center of the egg yolk, incubation for 3 days) and sodium arsenite 100 nM (injected into the center of the egg yolk, incubation for 3 days)	G1 vs. CG survival rate↑, body weight↑, relative extraembryonic vascular area↑, neural tube closure defects↓ whole embryo, brain, spine: fluorescence signals of Nanog↓, fluorescence intensity of SCP1↓, fluorescence signals of Tuj-1↑, fluorescence intensity of 5-mec↑ spinal cord: positive signal of Nanog↓, positive signal of SCP1↓, positive signal of 5-mec↑, % of survival cells↑ expression of: DNMT3a↑, DNMT1↑, Bcl-2↑, Bax↓, caspase-3↑ protein level of: DNMT3a↑, DNMT1↑
			G2 vs. CG survival rate↔, body weight↔, relative extraembryonic vascular area↔, neural tube closure defects↔ whole embryo, brain, spine: fluorescence signals of Nanog↔, fluorescence intensity of SCP1↔, fluorescence signals of Tuj-1↔, fluorescence intensity of 5-mec↔, % of MOD↔ spinal cord: positive signal of Nanog↔, positive signal of SCP1↔, positive signal of 5-mec↔, % of survival cells↔ expression of: DNMT3a↔, DNMT1↔, Bcl-2↔, Bax↔, caspase-3↔ protein level of: DNMT3a↔, DNMT1↔

↑—significant increase; ↓—significant decrease; ↔—no significant changes; [Ca^2+^]i—intracellular calcium; 5-mec—anti-5-methylcytidine; APD—action potential duration; Bax—BCL2-associated X protein; Bcl-2—B-cell lymphoma protein 2 alpha; CG—control group; DMA—dimethylarsinic acid; DNA—deoxyribonucleic acid; DNMT1—DNA methyltransferase 1; DNMT3a—DNA methyltransferases 3A; G1—group 1; G2—group 2; iAs—inorganic arsenic; ICa-L—L type calcium currents; IV—intravenous; MMA—monomethylarsonic acid; MN-PCE—micronuclei in polychromatic erythrocytes; MOD—mean optical densities; PCE—polychromatic erythrocyte; QTc—corrected QT interval; SCP1—Small C-terminal domain phosphatase 1; tAs—total arsenic species; Tuj-1—β3 Tubulin.

**Table 3:** Results of in vivo studies with iAs exposure and vitamin B_2_ treatment.

Reference	Research Model	Study Description	Main Results
Kumar et al., 2019 [19]	*Pangasianodon hypophthalmus*	CG (*n* = 6)—control diet (orally, for 95 days) and sodium arsenite in experimental water at 1/10th of LC_50_ (2.8 mg/L), (orally, added at 96 h) and temperature 34 °C (for 95 days) G1 (*n* = 6)—Se-NPs 0.5 mg/kg diet and vitamin B_2_ 5 mg/kg diet (orally, for 95 days) and sodium arsenite in experimental water at 1/10th of LC_50_ (2.8 mg/L), (orally, added at 96 h) and temperature 34 °C (for 95 days) G2 (*n* = 6)—Se-NPs 0.5 mg/kg diet and vitamin B_2_ 10 mg/kg diet (orally, for 95 days) and sodium arsenite in experimental water at 1/10th of LC_50_ (2.8 mg/L), (orally, added at 96 h) and temperature 34 °C (for 95 days) G3 (*n* = 6)—Se-NPs 0.5 mg/kg diet and vitamin B_2_ 15 mg/kg diet (orally, for 95 days) and sodium arsenite in experimental water at 1/10th of LC_50_ (2.8 mg/L), (orally, added at 96 h) and temperature 34 °C (for 95 days)	G1, 2, 3 vs. CG tAs: muscle↓ CTMin↓, LTMin↓, CTMax↑, LTMax↑ liver, gill, brain, kidney: CAT↓(during LTMin and LTMax) liver, gill, kidney: SOD↓ (during LTMin) brain: SOD↔ (during LTMin) liver: SOD↓ (during LTMax) gill, brain, kidney: SOD↔ (during LTMax) liver, gill, brain, kidney: GST↓, GPx↓ (during LTMin and LTMax) brain: AChE↑
Kumar et al., 2020 [20]	*Pangasianodon hypophthalmus*	CG (*n* = 6)—control diet (orally, for 90 days) and sodium arsenite in experimental water at 1/10th of LC_50_ (2.68 mg/L), (orally, added at 96 h) and temperature 34 °C (for 90 days) G1 (*n* = 6)—Se-NPs 0.5 mg/kg diet and vitamin B_2_ 5 mg/kg diet (orally, for 90 days) and sodium arsenite in experimental water at 1/10th of LC_50_ (2.68 mg/L), (orally, added at 96 h) and temperature 34 °C (for 90 days) G2 (*n* = 6)—Se-NPs 0.5 mg/kg diet and vitamin B_2_ 10 mg/kg diet (orally, for 90 days) and sodium arsenite in experimental water at 1/10th of LC_50_ (2.68 mg/L), (orally, added at 96 h) and temperature 34 °C (for 90 days) G3 (*n* = 6)—Se-NPs 0.5 mg/kg diet and vitamin B_2_ 15 mg/kg diet (orally, for 90 days) and sodium arsenite in experimental water at 1/10th of LC_50_ (2.68 mg/L), (orally, added at 96 h) and temperature 34 °C (for 90 days)	G1, 2, 3 vs. CG tAs: muscle↔, gill↓, kidney↓, brain↓ serum: cortisol↓, HSP 70↓, TP↑, albumin↓, globulin↑, A:G ratio↓, NBT↑, total immunoglobulin↑, myeloperoxidase↑ blood: glucose↓ liver, gill, brain, kidney: CAT↓, GST↓, GPx↓, LPO↓ liver, gill: SOD↓ brain, kidney: SOD↔ brain, muscle: AChE↑, vitamin C↑ FWG%↑, FER↑, PER↑, SGR↑, relative survival↑, cumulative mortality↓

↑—significant increase; ↓—significant decrease; ↔—no significant changes; A:G ratio—albumin globulin ratio; AChE—acetylcholine esterase; CAT—catalase; CG—control group; CTMax—critical thermal maximum; CTMin—critical thermal minimum; FER—feed efficiency ratio; FWG—final weight gain; G1—group 1; G2—group 2; G3—group 3; GPx—glutathione peroxidase; GST—glutathione-s-transferase; HSP 70—heat shock proteins 70; LC_50_—lethal concentration; LPO—lipid peroxidation; LTMax—lethal thermal maximum; LTMin—lethal thermal minimum; NBT—nitroblue tetrazolium; PER—protein efficiency ratio; Se-NPs—selenium nanoparticles; SGR—specific growth rate; SOD—superoxide dismutase; tAs—total arsenic species; TP—total protein.

**Table 4:** Results of in vivo studies with iAs exposure and vitamin B_12_ treatment.

Reference	Research Model	Study Description	Main Results
Majumdar et al., 2010 [21]	Rats, Albino, adult, male	CG (*n* = 6)—arsenic trioxide 3 mg/kg bw/day (orally, for 30 days) G1 (*n* = 6)—vitamin B_12_ 0.63 µg/kg bw/day (orally, for 30 days) and arsenic trioxide 3 mg/kg bw/day (orally, for 30 days)	G1 vs. CG urine: tAs↑ blood, liver: tAs↓ hepatic mitochondria: NO↓, TBARS↓, OH^−^↓, SOD↔, CAT↔, GSH↑ damaging changes in liver histology↓ liver: ALT↓, AST↓, ACP↓, iNOS↔ Mitochondrial Swelling↓, Mitochondrial Cytochrome c oxidase↑, Mitochondrial Calcium↑, Mitochondrial Ca^2+^-ATPase activity↑, Mitochondrial Caspase 3 activity↓ hepatic cell DNA smearing↓
Chen and Whanger 1994 [22]	Rats, Weanling and Sprague-Dawley, adult, male	CG (*n* = 5)—arsenite 0–150 µg/g (orally, for 8 weeks) G1 (*n* = 5)—vitamin B_12_ sufficient diet 100 µg/kg (orally, for 8 weeks) and arsenite 0–150 µg/g (orally, for 8 weeks)	G1 vs. CG liver: levels of MT↓
Acharyya et al., 2015 [23]	Rats, Albino, adult, female	CG (*n* = 6)—drinking water (orally, for 28 days) G1 (*n* = 6)—vitamin B_12_ 0.07 µg/100 g bw/day dissolved in water 200 µL/day (orally, for 28 days) and sodium arsenite 0.6 ppm/100 g bw/day (orally, for 28 days)	G1 vs. CG serum: ALP↔, AST↔, LDH↔, uric acid↔ hepato-somatic index↔, reno-somatic index↑ liver: MDA↔, XO↔, CAT↔ lung: CD↔, NPSH↔ intestine: MDA↔, CD↔, CAT↔

↑—significant increase; ↓—significant decrease; ↔—no significant changes; ACP—acid phosphatase; ALP—alkaline phosphatase; ALT—alanine aminotransferase; AST—aspartate amino transferase; Ca^2+^-ATPase—calcium adenosine triphosphatase; CAT—catalase; CD—conjugated diene; CG—control group; DNA—deoxyribonucleic acid; G1—group 1; GSH—glutathione; iNOS—inducible nitric oxide synthase; LDH—lactate dehydrogenase; MDA—malondialdehyde; MT—metallothionein; NO—nitric oxide; NPSH—nonprotein-soluble thiol; OH-—hydroxide; tAs—total arsenic species; TBARS—thiobarbituric acid reactive substances; XO—xanthine oxidase.

**Table 5:** Results of in vivo and in vitro studies with iAs exposure and folic acid treatment.

Reference	Research Model	Study Description	Main Results
Majumdar et al., 2010 [21]	Rats, Albino, adult, male	CG (*n* = 6)—arsenic trioxide 3 mg/kg bw/day (orally, for 30 days) G1 (*n* = 6)—folic acid 36 µg/kg bw/day (orally, for 30 days) and arsenic trioxide 3 mg/kg bw/day (orally, for 30 days)	G1 vs. CG urine: tAs↑ blood, liver: tAs↓ hepatic mitochondria: NO↓, TBARS↓, OH^−^↓, SOD↑, CAT↑, GSH↑ damaging changes in liver histology↓ liver: iNOS↓, ALT↓, AST↓, ACP↓ Mitochondrial Swelling↓, Mitochondrial Cytochrome c oxidase↑, Mitochondrial Calcium↑, Mitochondrial Ca^2+^-ATPase activity↑, Mitochondrial Caspase 3 activity↓ hepatic cell DNA smearing↓
Majumdar et al., 2009 [24]	Rats, Albino, adult, male	CG (*n* = 6)—arsenic trioxide 3 mg/kg bw/day (orally, for 30 days) G1 (*n* = 6)—folic acid 36 µg/kg bw/day (orally, for 30 days) and arsenic trioxide 3 mg/kg bw/day (orally, for 30 days)	G1 vs. CG urine: tAs↑ plasma and pancreatic islet cell mitochondria: NO↓, MDA↓, OH^−^↓, SOD↑, CAT↑, GSH↑ pancreatic islet cell mitochondria and lymphocyte: DNA smearing↓
Choudhry et al., 2009 [25]	Rats, Long Evans Norwegian Strains, adult, male	CG (*n* = 6)—arsenic 1 mg/L in drinking water (orally, for 2 weeks) G1 (*n* = 6)—folic acid 200 µg/day (orally, for 2 weeks) and arsenic 1 mg/L in drinking water (orally, for 2 weeks)	G1 vs. CG stool: bacterial count↓ tAs: in stool↓, in liver↓
Huang et al., 2018 [26]	Mice, C57BL/6J adult, male and female	CG (*n* = 16)—low-fat diet with folate 0.2 mg/kg/diet (orally, 6 weeks) for and arsenite 100 ppb in drinking water (orally, for 6 weeks) G1 (*n* = 16)—low-fat diet with folate 10 mg/kg/diet (orally, for 6 weeks) and arsenite 100 ppb (orally, for 6 weeks)	G1 vs. CG urine: tAs↔, %iAs↔, %DMAs↔ (in the male group) urine: tAs↔, %iAs↓, %DMAs↑ (in the female group)
	Mice, As3mt-KO, adult, male and female	CG (*n* = 16–20)—low-fat diet with folate 0.2 mg/kg/diet (orally, 6 weeks) for and arsenite 100 ppb in drinking water (orally, for 6 weeks) G1 (*n* = 16–20)—low-fat diet with folate 10 mg/kg/diet (orally, for 6 weeks) and arsenite 100 ppb in drinking water (orally, for 6 weeks)	G1 vs. CG urine: tAs↔, %DMAs (not detected), %MMAs (not detected)
	Mice, C57BL/6J adult, male and female	CG (*n* = 16)—low-fat diet with folate 0.2 mg/kg/diet (orally, for 24 weeks) and arsenite 100 ppb (orally, for 24 weeks) after that high-fat diet with folate 0.2 mg/kg/diet (orally, for 8 weeks) and arsenite 100 ppb in drinking water (orally, for 13 weeks) G1 (*n* = 16)—low-fat diet with folate 10 mg/kg/diet (orally, for 24 weeks) and arsenite 100 ppb in drinking water (orally, for 24 weeks) after that high-fat diet with folate 10 mg/kg/diet (orally, for 8 weeks) and arsenite 100 ppb in drinking water (orally, for 13 weeks)	G1 vs. CG FPI↔, HOMA-IR↔ (after 24 weeks on a low fat diet) FPI↓, HOMA-IR↓ (marginally significant, after 8 week on a high fat diet) liver: tAs↔, %iAs↔, %MMAs↔, %DMAs↔ (in the male group) liver: tAs↓, %iAs↓, %MMAs↑, %DMAs↑ (in the female group)
	Mice, As3mt-KO, adult, male and female	CG (*n* = 16–20)—low-fat diet with folate 0.2 mg/kg/diet (orally, for 24 weeks) and arsenite 100 ppb in drinking water (orally, for 24 weeks) after that high-fat diet with folate 0.2 mg/kg/diet (orally, for 8 weeks) and arsenite 100 ppb in drinking water (orally, for 13 weeks) G1 (*n* = 16–20)—low-fat diet with folate 10 mg/kg/diet (orally, for 24 weeks) and arsenite 100 ppb in drinking water (orally, for 24 weeks) after that high-fat diet with folate 10 mg/kg/diet (orally, for 8 weeks) and arsenite 100 ppb in drinking water (orally, for 13 weeks)	G1 vs. CG FPI↔, HOMA-IR↔ (after 24 weeks on a low fat diet and after 8 week on a high fat diet) liver: tAs↔
Tsang et al., 2012 [27]	Fetal mice (mice, CD1, adult, female)	CG (*n* = 12)—control diet with folate 2.2 mg/kg/diet (orally, from gestation day 1 to 18) and sodium meta-arsenite 85 ppm in drinking water (orally, from gestation day 8 to 18) G1 (*n* = 12)—control diet with folate 11.0 mg/kg/diet (orally, from gestation day 5 to 18) and sodium meta-arsenite 85 ppm in drinking water (orally, from gestation day 8 to 18)	G1 vs. CG maternal livers: iAs↓, methylation ratios of DMAs/MAs↑, methylation ratios of (MAs+DMAs)/iAs↑ fetal livers: tAs↔, speciated As↔, SAH↑, SAM/SAH↔, mRNA level of Dnmt3↔ body weights of fetuses↓ in G1: changed the CpG island methylation including genes associated with cancer and fetal development, altered methylation status of genes involved in the Wnt-signaling pathway
Spiegelstein et al., 2003 [28]	Mice, Folbp1^+^/^+^, adult, male	CG (*n* = 5)—control diet with folate 2.7 mg/kg/diet (orally, for 20 days) and sodium arsenate 10 µL/g bw (IP injection, once dose) G1 (*n* = 5)—control diet with folate 2.7 mg/kg/diet (orally, for 20 days) and sodium arsenate 10 µL/g bw (IP injection, once dose) after that folate deficient diet 0.3 mg/kg/diet (orally, for 27 days)	G1 vs. CG urine: %As(V)↔, %As(III)↔, MMA(V), (not detected), %DMA(V)↔, %tAs↓ plasma: folate↓, SAM↔, SAH↔, SAM/SAH↔
	Mice, Folbp1^−^/^−^, adult, male	CG (*n* = 6)—control diet with folate 2.7 mg/kg/diet (orally, for 20 days) and sodium arsenate 10 µL/g bw (IP injection, once dose) G2 (*n* = 6)—control diet with folate 2.7 mg/kg/diet (orally, for 20 days) and sodium arsenate 10 µL/g bw (IP injection, once dose) after that folate deficient diet 0.3 mg/kg/diet (orally, for 27 days)	G2 vs. CG urine: %As(V)↔, %As(III)↔, MMA(V), (not detected), %DMA(V)↔, %tAs↔ plasma: folate↓, SAM↔, SAH↔, SAM/SAH↔
			G2 vs. G1 urine: %As(V)↓, %As(III)↔, MMA(V), (not detected), %DMA(V)↑, %tAs↔ plasma: folate↓, SAM↔, SAH↔, SAM/SAH↔
Spiegelstein et al., 2005 [29]	Mice, wild type, adult, male	CG (*n* = 5)—control diet with folate 2.7 mg/kg/diet (orally, for 20 days) and sodium arsenate 10 µL/g bw (IP injection, once dose) G1 (*n* = 5)—control diet with folate 2.7 mg/kg/diet (orally, for 20 days) and sodium arsenate 10 µL/g bw (IP injection, once dose) after that folate deficient diet 0.3 mg/kg/diet (orally, for 27 days)	G1 vs. CG urine: %As(V)↔, %As(III)↔, %MMA(V), (not detected), %DMA(V)↔, %tAs↓ plasma: folate↓, SAH↔, SAM↔, SAM/SAH↔
	Mice, Folbp2^−^/^−^, adult, male	CG (*n* = 5)—control diet with folate 2.7 mg/kg/diet (orally, for 20 days) and sodium arsenate 10 µL/g bw (IP injection, once dose) G2 (*n* = 5)—control diet with folate 2.7 mg/kg/diet (orally, for 20 days) and sodium arsenate 10 µL/g bw (IP injection, once dose) after that folate deficient diet 0.3 mg/kg/diet (orally, for 27 days)	G2 vs. CG urine: %As(V)↔, %As(III)↔, %MMA(V), (not detected), %DMA(V)↔, %tAs↔ plasma: folate↓, SAH↑, SAM↔, SAM/SAH↓
			G2 vs. G1 urine: %As(V)↔, %As(III)↔, %MMA(V), (not detected), %DMA(V)↔, %tAs↔ plasma: folate↓, SAH↑, SAM↓, SAM/SAH↓
Acharyya et al., 2015 [23]	Rats, Albino, adult, female	CG (*n* = 6)—drinking water (orally, for 28 days) G1 (*n* = 6)—folic acid 4 µg/100 g bw/day dissolved in water 200 µL/day (orally, for 28 days) and sodium arsenite 0.6 ppm/100 g bw/day (orally, for 28 days)	G1 vs. CG serum: ALP↔, ALT↔, urea↔ hepato-somatic index↔, reno-somatic index↑ liver: MDA↔, XO↔, CAT↔
Mukherjee et al., 2006 [30]	Rats, Albino, adults, male	CG (*n* = 5)—arsenic trioxide 3 mg/kg bw/day (orally, for 30 days) G1 (*n* = 5)—folic acid 36 µg/kg bw/day (orally, for 30 days) and arsenic trioxide 3 mg/kg bw/day (orally, for 30 days)	G1 vs. CG pancreatic tissue: NO↓, MDA↓, OH^−^↓, SOD↑, GSH↑, CAT↑ serum: TNF-α↔, IL-6↔ islet cell counts↑
McDorman et al., 2002 [31]	Mice, C57Bl/6J, adult, male	CG (*n* = 5)—folate deficient diet (orally, for 7 weeks) and sodium arsenite 0, 2.5, 5, 10 mg/kg bw/day (orally, during week 7 for 4 days at 24 h intervals) G1 (*n* = 5)—folic acid 5 mg/kg diet (orally, for 7 weeks) and sodium arsenite 0, 2.5, 5, 10 mg/kg bw/day (orally, during week 7 for 4 days at 24 h intervals)	G1 vs. CG after 10 mg/kg bw/day sodium arsenite: RBC folate↑, MN-PCEs/1000↓, MN-NCEs↓
Lin et al., 2018 [32]	Fetal rats (Rats, Sprague Dawley, adult, female)	CG (*n* = 10)—sodium arsenic 75 mg/L in drinking water (orally, for 6 weeks) G1 (*n* = 10)—folate 0.53 mg/kg bw/day (orally, for 6 weeks) and sodium arsenic 75 mg/L in drinking water (orally, for 6 weeks) G2 (*n* = 10)—folate 5.3 mg/kg bw/day (orally, for 6 weeks) and sodium arsenic 75 mg/L in drinking water (orally, for 6 weeks) G3 (*n* = 10)—folate 10.6 mg/kg bw/day (orally, for 6 weeks) and sodium arsenic 75 mg/L in drinking water (orally, for 6 weeks)	G1 vs. CG weight of fetus↔, weight of placenta↔, heart malformation↔ embryonic heart: relative mRNA level and relative protein level of: NkX2.5↔, GATA-4↔, TBX5↑
			G2 vs. CG weight of fetus↑, weight of placenta↑, heart malformation↔ embryonic heart: relative mRNA level of: NkX2.5↑, GATA-4↑, TBX5↑ embryonic heart: relative protein level of: NkX2.5↑, GATA-4↑, TBX5↔
			G3 vs. CG weight of fetus↑, weight of placenta↑, heart malformation↔ embryonic heart: relative mRNA level and relative protein level of: NkX2.5↑, GATA-4↑, TBX5↑
Na et al., 2020 [33]	Fetal rats (Rats, Sprague Dawley, adult, female)	CG (*n* = 12)—sodium arsenic 75 mg/L in drinking water (orally, for 6 weeks) G1 (*n* = 12)—folic acid 0.53 mg/kg bw/day (orally, for 6 weeks) and sodium arsenic 75 mg/L in drinking water (orally, for 6 weeks) G2 (*n* = 12)—folic acid 5.3 mg/kg bw/day (orally, for 6 weeks) and sodium arsenic 75 mg/L in drinking water (orally, for 6 weeks) G3 (*n* = 12)—folic acid 10.6 mg/kg bw/day (orally, for 6 weeks) and sodium arsenic 75 mg/L in drinking water (orally, for 6 weeks)	G1 vs. CG weight of fetus↓, weight of placenta↔, heart malformation↓ fetal heart: mRNA expression levels of Mef2C↓, levels of H3AcK9↓
			G2 vs. CG weight of fetus↑, weight of placenta↑, heart malformation↓ fetal heart: mRNA expression levels of Mef2C↓, levels of H3AcK9↓
			G3 vs. CG weight of fetus↑, weight of placenta↑, heart malformation↓ fetal heart: mRNA expression levels of Mef2C↓, levels of H3AcK9↓
Gefrides et al., 2002 [34]	Fetal mice (Mice, heterozygotus Splotch, adult, female)	CG—arsenic acid 40 mg/kg bw/day (IP injection, once on each of gestational day 7.5 and 8.5) and sacrificed on gestational day 18.5 G1—folic acid 25 mg/kg bw/day (IP injection, gestational day 6.5 and 10.5) and arsenic acid 40 mg/kg bw/day (IP injection, once on each of gestational day 7.5 and 8.5) and sacrificed on gestational day 18.5 G2—folinic acid 2 mg/kg bw/day (IP injection, from gestational day 6.5 and 10.5) and arsenic acid 40 mg/kg bw/day (IP injection, once on each of gestational day 7.5 and 8.5) and sacrificed on gestational day 18.5	G1 vs. CG maternally lethal↑
			G2 vs. CG NTDs↔
	Fetal mice (Mice wild-type Splotch, adult, female)	CG—arsenic acid 40 mg/kg bw/day (IP injection, once on each of gestational day 7.5 and 8.5) and sacrificed on gestational day 18.5 G1—folic acid 25 mg/kg bw/day (IP injection, gestational day 6.5 and 10.5) and arsenic acid 40 mg/kg bw/day (IP injection, once on each of gestational day 7.5 and 8.5) and sacrificed on gestational day 18.5 G2—folinic acid 2 mg/kg bw/day (IP injection, from gestational day 6.5 and 10.5) and arsenic acid 40 mg/kg bw/day (IP injection, once on each of gestational day 7.5 and 8.5) and sacrificed on gestational day 18.5	G1 vs. CG maternally lethal↑
			G2 vs. CG NTDs↓ embryo/fetal lethality↑
	Fetal mice (Mice, LM/Bc and SWV litters, adult, female)	CG—arsenic acid 40 mg/kg bw/day (IP injection, once on each of gestational day 7.5 and 8.5) and sacrificed on gestational day 18.5 G1—folic acid 25 mg/kg bw/day (IP injection, gestational day 6.5 and 10.5) and arsenic acid 40 mg/kg bw/day (IP injection, once on each of gestational day 7.5 and 8.5) and sacrificed on gestational day 18.5 G2—folinic acid 2 mg/kg bw/day (IP injection, from gestational day 6.5 and 10.5) and arsenic acid 40 mg/kg bw/day (IP injection, once on each of gestational day 7.5 and 8.5) and sacrificed on gestational day 18.5	G1 vs. CG maternally lethal↑
			G2 vs. CG NTDs↔ embryo/fetal lethality↔
Wlodarczyk et al., 2001 [35]	Fetal mice (Mice, Folbp2^−^/^−^, adult, female)	CG (*n* = 6)—control diet with folate 2.7 mg/kg (orally, for 2–3 weeks prior to the first attempts at mating and for all pregnancy) and sodium arsenate 40 mg/kg (IP injection on gestational days 7.5 and 8.5) G1 (*n* = 6)—folate deficient diet 0.3 mg/kg (orally, for 2–3 weeks prior to the first attempts at mating and for all pregnancy) and sodium arsenate 40 mg/kg (IP injection on gestational days 7.5 and 8.5)	G1 vs. CG rate of exencephaly↑ G2 vs. CG rate of exencephaly↔ G1 vs. G2 rate of exencephaly↑
	Fetal mice (Mice, Folbp2^+^/^+^, adult, female)	G2 (*n* = 6)—folate deficient diet 0.3 mg/kg/diet (orally, for 2–3 weeks prior to the first attempts at mating and for all pregnancy) and sodium arsenate 40 mg/kg (IP injection on gestational days 7.5 and 8.5)	
Ma et al., 2015 [36]	Wild-type AB strain and Tg (cmlc2:GFP) zebrafish, embryos	CG—sodium arsenite 2 mM G1—folic acid 50 µM and sodium arsenite 2 mM G2—folic acid 100 µM and sodium arsenite 2 mM	G1, G2 vs. CG after 96 h: hatched embryos↑, survival↑, affected embryos↓
			G2 vs. CG abnormal development↓, ventricle development↑, cardiac looping↑, normal erythropoiesis↑, axons in all areas of the brain↑, mRNA level of Dvr1↑
Dubey and Shea 2007 [37]	NB2a/d1 cells	CG—sodium arsenite 0.07 µm (for 24 h) G1—absence folate and sodium arsenite 0.07 µm (for 24 h)	G1 vs. CG neurofilament transport↓ perikaryal RT97↑ perikaryal phospho-NF immunoreactivity↑
Nelson et al., 2007 [38]	Mice, C57BL/6, adult	CG (*n* = 4)—folate sufficient diet 5 mg/kg (orally, for 30 days) and sodium arsenite 1 ppm in drinking water (orally, for 30 days) G1 (*n* = 4)—folate deficient diet (orally, for 30 days) and sodium arsenite 1 ppm in drinking water (orally, for 30 days)	G1 vs. CG serum: folate↓ blood: homocysteine↑ zinc-finger transcription factors↓ expression of epidermal development and differentiation (hair and skin) genes↓ expression of cellular movement genes↑
Ruan et al., 2000 [39]	Fibroblasts embryo, (SWV/Fnn, adult, female)	CG—folic acid to final concentration 9 µM (for 24 h) and sodium arsenite 10 µM (for 24 h) G1—folic acid to final concentration 270 µM (for 24 h) and sodium arsenite 10 µM (for 24 h)	G1 vs. CG viability↑
		G1—folic acid to final concentration 100 µM (for 24 h) and sodium arsenite 6 or 10 µM (for 24 h)	in the G1: viability↔
		CG—folic acid to final concentration 9 µM and sodium arsenite 1 µM G1—folic acid to final concentration 90 µM and sodium arsenite 1 µM	G1 vs. CG at day 6: number of cells↔ at day 7: number of cells↑
		CG—folic acid to final concentration 9 µM (for 24 h) and dimethylarsinic acid 10 mM (for 24 h) G1—folic acid to final concentration 270 µM (for 24 h) and dimethylarsinic acid 10 mM (for 24 h)	G1 vs. CG viability↑
		G1—folic acid to final concentration 100 µM (for 24 h) and dimethylarsinic acid 3 or 10 mM (for 24 h)	in the G1: viability↔
Crandall and Vorce 2002 [40]	Fibroblast Folbp2^−^/^−^	CG—sodium arsenite 100 µM (for 24 h) G1—folic acid 30 µM (for 1 week) and after that sodium arsenite 100 µM (for 24 h)	G1 vs. CG survival↔
		CG—sodium arsenite 0.3 or 3 µM G1—folic acid 7 nM (for 2 h) and sodium arsenite 0.3 or 3 µM	G1 vs. CG folic acid uptake↓
	Fibroblast Folbp2^+^/^+^	CG—sodium arsenite 100 µM (for 24 h) G1—folic acid 30 µM (for 1 week) and after that sodium arsenite 100 µM (for 24 h)	G1 vs. CG survival↑
		CG—sodium arsenite 0.3 or 3 µM G1—folic acid 7 nM (for 2 h) and sodium arsenite 0.3 or 3 µM	G1 vs. CG folic acid uptake↔

↑—significant increase; ↓—significant decrease; ↔—no significant changes; ACP—acid phosphatase; ALP—alkaline phosphatase; ALT—alanine aminotransferase; As(III)—arsenite; As(V)—arsenate; AST—aspartate amino transferase; Ca^2+^-ATPase—calcium adenosine triphosphatase; CAT—catalase; CG—control group; DMA(V)—dimethylarsonic acid; DMAs—dimethylarsenic; DMG—dimethylglycine; DNA—deoxyribonucleic acid; Dnmt3—DNA(cytosine-5)-methyltransferase 3-alpha; Dvr1—decapentaplegic and Vg-related-1; Folbp—folate binding proteins; FPI—fasting plasma insulin; G1—group 1; G2—Group 2; G3—group 3; GSH—glutathione; H3AcK9—acetytalion of histone H3 lysine 9; IL-6—Interleukin 6; iNOS—inducible nitric oxide synthase; IP—intraperitoneally; MAs—monomethylarsenic; MDA—malondialdehyde; Mef2C—myocyte enhancer factor-2C; MMA(V)—monomethylarsonic acid; MN—micronuclei; NCEs—normochromatic erythrocytes; NF—neurofilament; NO—nitric oxide; NTDs—neural tube defects; OH-—hydroxide; PCEs—polychromatic erythrocytes; RBC—red blood cell; SAH—S-adenosylhomocysteine; SAM—S-adenosylmethionine; SOD—superoxide dismutase; tAs—total arsenic species; TBARS—thiobarbituric acid reactive substances; TNF-α—tumor necrosis factor-α; XO—xanthine oxidase.

**Table 6:** Results of in vivo studies with iAs exposure and simultaneous vitamin B_12_ and folic acid treatment.

Reference	Research Model	Study Description	Main Results
Majumdar et al., 2010 [21]	Rats, Albino, adult, male	CG (*n* = 6)—arsenic trioxide 3 mg/kg bw/day (orally, for 30 days) G1 (*n* = 6)—vitamin B_12_ 0.63 µg/kg bw/day, folic acid 3 mg/kg bw/day (orally, for 30 days) and arsenic trioxide 3 mg/kg bw/day (orally, for 30 days)	G1 vs. CG urine: tAs↑ blood, liver: tAs↓ hepatic mitochondria: NO↓, TBARS↓, OH^−^↓,SOD↑, CAT↑, GSH↑ damaging changes in liver histology↓ liver: iNOS↓, ALT↓, AST↓, ACP↓ Mitochondrial Swelling↓, Mitochondrial Cytochrome c oxidase↑, Mitochondrial Calcium↑, Mitochondrial Ca^2+-^ATPase activity ↑, Mitochondrial Caspase 3 activity↓ hepatic cell DNA smearing↓
Majumdar et al., 2009 [24]	Rats, Albino, adult, male	CG (*n* = 6)—arsenic trioxide 3 mg/kg bw/day (orally, for 30 days) G1 (*n* = 6)—vitamin B_12_ 0.63 µg/kg bw/day, folic acid 36 µg/kg bw/day (orally, for 30 days) and arsenic trioxide 3 mg/kg bw/day (orally, for 30 days)	G1 vs. CG urine: tAs↑ plasma and pancreatic islet cell mitochondria: NO↓, MDA↓, OH^−^↓, SOD↑, CAT↑, GSH↑ pancreatic islet cell mitochondria and lymphocyte: DNA smearing↓
Huang et al., 2018 [41]	Mice, C57BL/6J, adult, female	CG (*n* = 6)—vitamin B_12_ 10 µg/kg/diet, folate 2 mg/kg/diet (adequate diet) and deionized water (orally, for 1 week) after that adequate diet and sodium arsenite 100 or 1000 ppb in drinking water (orally, for 1 week) after that mating for 1 week and diet and exposure (the same that before mating) until parturition, after giving birth vitamin adequate diet and deionized water G1 (*n* = 6)—vitamin B_12_ 10 µg/kg/diet, folate 2 mg/kg/diet and deionized water (orally, for 1 week) and after that vitamin B_12_ 50 µg/kg/diet, folate 6 mg/kg/diet (supplemented diet) and sodium arsenite 100 or 1000 ppb in drinking water (orally, for 1 week) after that mating for 1 week and diet and exposure (the same that before mating) until parturition after giving birth adequate diet and deionized water	G1 vs. CG in the group with 100 ppb sodium arsenite: urine: MAs↑, DMAs↑, iAs↔, tAs↔ in the group with 1000 ppb sodium arsenite: urine: MAs↔, DMAs↔, iAs↔, tAs↔
	Mice, C57BL/6J, offspring, male	CG (*n* = 5–16)—prenatally exposed to adequate diet and sodium arsenite 100 or 1000 ppb in drinking water G1 (*n* = 5–16)—prenatally exposed to supplemented diet and sodium arsenite 100 or 1000 ppb in drinking water	G1 vs. CG in the group with 100 ppb sodium arsenite: 13-week-old: AUC↓ (glucose tolerance test) 14-week- old: FPI↓, HOMA-IR↓ in the group with 1000 ppb sodium arsenite: 14-week- old: FPI↓, HOMA-IR↓ liver: fraction of methylated DNA↑
	Mice, C57BL/6J, offspring, female	CG (*n* = 5–16)—prenatally exposed to adequate diet and sodium arsenite 100 or 1000 ppb in drinking water G1 (*n* = 5–16)—prenatally exposed to supplemented diet and sodium arsenite 100 or 1000 ppb in drinking water	G1 vs. CG 14-week- old: FPI↔, HOMA-IR↔
Chattopadhyay et al., 2012 [42]	Rats, Wistar, adult, female	CG (*n* = 6)—sodium arsenite 0.4 ppm/100 g bw/day (orally, for 24 days) G1 (*n* = 6)—vitamin B_12_ 0.07 µg with folic acid 4.0 µg dissolved in 0.1 mL of distilled water/100 g bw (by gavage, for 24 days) and sodium arsenite 0.4 ppm/100 g bw/day (orally, for 24 days)	G1 vs. CG hepatosomatic index↓ hepatic histoarchitecture↑ liver: ALT↓, AST↓, TP↑, MDA,↓ CD↓, SOD↑, CAT↑, NPSH↑, DNA fragmentation↓ plasma: TCH↓, TG↓, LDL↓, HDL↑
Mukherjee et al., 2006 [30]	Rats, Albino, adults, male	CG (*n* = 5)—arsenic trioxide 3 mg/kg bw/day (orally, for 30 days) G1 (*n* = 5)—vitamin B_12_ 0.63 µg/kg bw/day with folic acid 36 µg/kg bw/day (orally, for 30 days) and arsenic trioxide 3 mg/kg bw/day (orally, for 30 days)	G1 vs. CG pancreatic tissue: NO↓, MDA↓, OH^−^↓, SOD↑, GSH↑, CAT↑ serum: TNF-α↓, IL-6↓ islet cell counts↑
Acharyya et al., 2015 [23]	Rats, Albino, adult, female	CG (*n* = 6)—drinking water (orally, for 28 days) G1 (*n* = 6)—vitamin B_12_ 0.07 µg/100 g bw/day with folic acid 4.0 µg/100 g bw/day dissolved in water 200 µL/day (orally, for 28 days) and sodium arsenite 0.6 ppm/100 g bw/day (orally, for 28 days)	G1 vs. CG serum: ALP↔, AST↔, ALT↔, LDH↔, uric acid↔, urea↔, creatinine↔ liver: MDA↔, NPSH↔, CAT↔, XO↔ lung: CD↔ intestine: MDA↔, CD↔, CAT↔ liver and kidney: DNA breakage↓ hepatic and renal histoarchitecture↑, reno-somatic index↑

↑—significant increase; ↓—significant decrease; ↔—no significant changes; ACP—acid phosphatase; ALP—alkaline phosphatase; ALT—alanine aminotransferase; AST—aspartate amino transferase; AUC—area under the curve; CAT—catalase; CD—conjugated diene; CG—control group; DMAs—dimethylarsenic; DNA—deoxyribonucleic acid; FPI—fasting plasma insulin; G1—group 1; GSH—glutathione; HDL—high-density lipoprotein; HOMA-IR—homeostasis model assessment-insulin resistance; iAs—inorganic arsenic; IL-6—Interleukin 6; iNOS—inducible nitric oxide synthase; LDH—lactate dehydrogenase; LDL—low-density lipoprotein; MAs—monomethylarsenic; MDA—malondialdehyde; NO—nitric oxide; NPSH—nonprotein-soluble thiol; OH^−^—hydroxide; SOD—superoxide dismutase; tAs—total arsenic species; TBARS—thiobarbituric acid reactive substances; TCH—total cholesterol; TG—triglyceride; TNF-α—tumor necrosis factor-α; TP—total protein; XO—xanthine oxidase.

**Table 7:** Results of in vivo and in vitro studies with iAs exposure and zinc treatment.

Reference	Research Model	Study Description	Main Results
Kumar et al., 2011 [43]	Rats, Wistar, adult, male	CG (*n* = 8)—sodium arsenite 100 ppm in drinking water (orally, for 3 months) G1 (*n* = 8)—zinc sulfate 227 mg/L in drinking water (orally, for 3 months) and sodium arsenite 100 ppm in drinking (orally, for 3 months)	G1 vs. CG liver: tAs↓, Zn↑ % uptake values of ^65^Zn: brain↓, liver↓, kidney↔, intestine↔, spleen↔, lungs↔ biological half-lives slow and fast component of ^65^Zn in whole body↔ biological half-lives fast component of ^65^Zn in liver↓
Wang et al., 2020 [44]	Cyprinus carpio	CG (*n* = 30)—arsenic trioxide 2.83 mg/L (orally, for 1 month) G1 (*n* = 30)—zinc chloride 1 mg/L and (orally, for 1 month) and arsenic trioxide 2.83 mg/L (orally, for 1 month)	G1 vs. CG kidney: tAs↓, Zn↔, CAT↑ protein levels of: HSP60↓, HSP70↓, HSP90↓, Beclin-1↑, LC3↓, p62↑, GRP78↔, p-PERK↓, p-eIF2a↔, pI3K↔, p-AKT↑, p-mTOR↑ mRNA levels of: Occludin↑, ZO-1↑, ZO-2↑, Claudin 3↑, Claudin 4↑, Claudin 7↑, Claudin 11↔, Claudin 15↔, GRP78↓, ATF-6↓, IRE1↓, CHOP↓, MT↑, ZnT1↑, ZnT5↑, ZIP7↑, ZIP8↑, ZIP10↑
Wang et al., 2021 [45]	Cyprinus carpio	CG—arsenic 2.83 mg/L G1—zinc 1 mg/L and arsenic 2.83 mg/L	G1 vs. CG spleen tissues: tAs↓, Ca↓, Bax/Bcl-2 ratio↓, LC3-II/LC3-I ratio↓, mRNA levels of: ATPα↑, Na^+^/K^+^-ATPase↑, Ca^2+^-Mg^2+^-ATPase↑, GRP78↓, GRP94↓, PERK↓, eIF2α↔, IRE1↔, ATF6↔, CHOP↓, Fas↓, caspase 8↓, caspase 9↓, caspase 3↓, Bax↓, Bcl-2↑, Beclin1↓, ATG-5↓, p62↑, LC3-I↔, LC3-II↓, protein levels of: caspase-3↓, p-eIF2α↓, p-PERK↓, p62↑, Beclin1↓ changes in spleen tissues (apoptosis, endoplasmic reticulum damage)↓
Kumar and Reddy 2017 [46]	Rats, Wistar, young, adult, old, male	CG (*n* = 6)—sodium arsenite 10 mg/kg bw/day (orally, for 1 week) G1 (*n* = 6)—zinc chloride 0.02% through drinking deoinized water (orally, for 1 week) and sodium arsenite 10 mg/kg bw/day (orally, for 1 week)	G1 vs. CG cerebral cortex, cerebellum, hippocampus: tAs↓, level of AChE↓, activity of AChE↑ open field behavioral tasks↑, total locomotor activity↑, exploratory behavior↑, grip strength↑, behavioral assessments on water maze↑
Kadeyala et al., 2013 [47]	Rats, Wistar, 3 months old, 21 and 28 postnatal days	CG (*n* = 6)—sodium arsenite 100 ppm in sterile distilled water (orally, from gestation day 6 to 21 postnatal day) G1 (*n* = 6)—zinc 10 ppm in sterile distilled water (orally, from gestation day 6 to 21 postnatal day) and sodium arsenite 100 ppm in sterile distilled water (orally, from gestation day 6 to 21 postnatal day)	G1 vs. CG cerebral cortex, cerebellum, hippocampus: tAs↓, Mn-SOD↑, Cu/Zn-SOD↑, CAT↑, GPx↑, GR↑, GST↓, MDA↓, mRNA expression of: caspase 3↓, caspase 9↓
Modi et al., 2006 [48]	Rats, Wistar, adult, male	CG (*n* = 6)—sodium arsenite 2 mg/kg bw/day (orally, for 3 weeks) G1 (*n* = 6)—zinc sulfate 5 mg/kg bw/day (orally, for 3 weeks) and sodium arsenite 2 mg/kg bw/day (orally, for 3 weeks)	G1 vs. CG blood: tAs↔, Zn↔, ALAD↑ serum: ALT↓, AST↓ liver: tAs ↔, Zn↔, GSH↔, TBARS↓, CAT↓, ALP↑, ACP↔ kidney: tAs↔, Zn↔, GSH↔, TBARS↔, CAT↔
Modi et al., 2005 [49]	Mice, Swiss, adult, male	CG (*n* = 5)—sodium arsenite 2 mg/kg bw/day (IP, for 5 days) G1 (*n* = 5)—zinc acetate 10 mg/kg bw/day (orally, 2 h after arsenic for 5 days) and sodium arsenite 2 mg/kg bw/day (IP, for 5 days)	G1 vs. CG blood: tAs↔, Zn↔, ALAD↑, GSH↔, ZPP↔ liver: tAs↔, Zn↔, GSH↔, GSSG↓, TBARS↓ kidney: tAs↔, Zn↔, GSH↔, GSSG↔, TBARS↓
		CG (*n* = 5)—sodium arsenite 2 mg/kg bw/day (IP, for 5 days) after that saline (orally, for 3 days) G1 (*n* = 5)—sodium arsenite 2 mg/kg bw/day (IP, for 5 days) after that zinc acetate 10 mg/kg bw/day (orally, for 3 days)	G1 vs. CG blood: tAs↔, Zn↔, ALAD↔, GSH↔, ZPP↔ liver: tAs↔, Zn↔, GSH↔, GSSG↔, TBARS↔ kidney: tAs↔, Zn↔, GSH↔, GSSG↔, TBARS↔
Kreppel et al.1994 [50]	Mice, CF1, adult, male	CG (*n* = 6)—saline (sc injected, one dose) and after that arsenite-73 115 or 85 µmol/kg bw (sc injected, 24 h after, one dose) G1 (*n* = 6)—zinc acetate 1000 µmol/kg bw (sc injected, one dose) and after that arsenite-73 115 or 85 µmol/kg bw (sc injected, 24 h after zinc, one dose)	in the group with dose 115 µmol/kg bw—arsenic-73: liver↔, blood↓, kidney↓, skin↓, heart↓, brain↔, lung↓, small intestine↓, large intestine↔, muscle↓ in the group with 85 µmol/kg bw—arsenic-73: liver (nuc↔, Mit↔, Mic↑, Cyt↓), kidney (nuc↔, Mit↔, Mic↔, Cyt↔), small intestine (nuc↔, Mit↑, Mic↑, Cyt↔) arsenic-73 bound to MT↔
		CG (*n* = 15)—sodium arsenite 130 µmol/kg bw (sc injected, one dose) G1 (*n* = 15)—zinc acetate 100 µmol/kg bw (sc injected, one dose) and sodium arsenite 130 µmol/kg bw (sc, 96 h after zinc, one dose)	G1 vs. CG at 96 h: survival↑
		CG (*n* = 20–40)—saline (sc injected, one dose) and after that sodium arsenite 130 µmol/kg bw (sc injected, 24 h after, one dose) G1 (*n* = 20–40)—zinc acetate 1000 µmol/kg bw (sc injected, one dose) and sodium arsenite 130 µmol/kg bw (sc injected, 96 h after zinc, one dose)	G1 vs. CG correlation between MT induction and protection against the lethal effects of arsenic↔
Wang and Lee 1993 [51]	SA7N cells	CG—sodium arsenite 200 µM (for 120 min) G1—zinc sulfate 200 µM (for 24 h) and after that sodium arsenite 200 µM (for 120 min)	G1 vs. CG tAs: accumulation↓
		CG—sodium arsenite 200 µM (for 30 min) G1—zinc sulfate 200 µM (for 24 h) and after that sodium arsenite 200 µM (for 30 min) and after that incubated in normal medium (for 120 min)	G1 vs. CG tAs: excrection↑
Zhao et al., 2019 [52]	Cyprinus carpio	CG (*n* = 30)—arsenic trioxide 2.83 mg/L (orally, for 1 month) G1 (*n* = 30)—zinc 1 mg/L and (orally, for 1 month) and arsenic trioxide 2.83 mg/L (orally, for 1 month)	G1 vs. CG anterior, mid intestines: SOD↑, mRNA levels of: IL-1β↓, IL-6↓, IL-8↔, phosphorylation of IĸB-α↓, NF-ĸB nuclear translocation↓ histological changes in intestines↓ anterior intestines: mRNA levels of: Occludin↑, Claudin 3↑, Claudin 4↑, Claudin 7↑, Claudin 11↑, Claudin 15↑, ZO-1↑, ZO-2↑ mid intestines: mRNA levels of: Occludin↔, Claudin 3↔, Claudin 4↔, Claudin 7↑, Claudin 11↑, Claudin 15↑, ZO-1↑, ZO-2↑
Zhao et al., 2019 [53]	Cyprinus carpio	CG (*n* = 30)—arsenic trioxide 2.83 mg/L (orally, for 1 month) G1 (*n* = 30)—zinc 1 mg/L and (orally, for 1 month) and arsenic trioxide 2.83 mg/L (orally, for 1 month)	G1 vs. CG liver: ROS↓, Na^+^/K^+^-ATPase↑, Ca^2+^-Mg^2+^-ATPase↑, AST↑, ALT↑, ALP↓ activity of CYP1A↑, protein and mRNA levels of CYP1A↑, capase 3↓, caspase 8↓, Bax↓, Bcl-2↑, TNF-α↓, Fas↓ damage in nucleus and mitochondria↓
Ganger et al., 2016 [54]	Rats, Sprague Dawley, adult, male	CG (*n* = 6)—sodium arsenite 75 µmol/kg (sc injected, for 1 day) G1 (*n* = 6)—zinc sulfate 153 µmol/kg (sc injected, for 1 day) and sodium arsenite 75 µmol/kg (sc injected, for 1 day) after that zinc sulfate 153 µmol/kg (sc injected, for 1 day)	G1 vs. CG liver: MDA↓, GSH↑, NADPH↑, CAT↔, SOD↓, ALP↑, protein expression of MT↑, mRNA expression of MT-1↑, iron↑, Zn↑, potassium↓, chlorine↑, sulfur↑ histopathological changes (cytoplasmic vacuolization, sinusoidal expansions)↓
Wong et al., 2019 [55]	Mice, C57Bl/6, adult, female)	CG (*n* = 7)—zinc carbonate 30 mg/kg/diet (orally, for 6 weeks) and sodium arsenite 50 or 500 ppb in drinking water (orally, for 6 weeks) G1 (*n* = 7)—zinc carbonate 6 mg/kg/diet (orally, for 6 weeks) and sodium arsenite 50 or 500 ppb in drinking water (orally, for 6 weeks)	G1 vs. CG plasma: Zn↓ liver: transcript abundance of: HO-1↑, IL6↑, Ccl2↑, ICAM1↑
Cao et al., 2019 [56]	INS-1 (rat insulinoma pancreatic beta cells)	CG—zinc sulfate 4 µM (for 5 days) and after that sodium arsenite 50 ppm (for 24 h) G1—zinc sulfate 0 µM (for 5 days) and after that sodium arsenite 50 ppm (for 24 h)	G1 vs. CG Zn↓, calcium↔, copper↔, selenium↔, iron↔, magnesium↔, viable cells↓, % dead cells↔, insulin↔ mRNA level of: cleaved PARP↔, BAX/Bcl-2↔, Mt1↓, Mt2↔, HO-1↔, Ogg1↔, p53↔, 8-OHdG↔, ɣ-H2AX↔, Ins1↑, Pdx1↓, Neurod1↔, Znt8↔
		CG—zinc sulfate 4 µM (for 5 days) and after that sodium arsenite 500 ppm (for 24 h) G1—zinc sulfate 0 µM (for 5 days) and after that sodium arsenite 500 ppm (for 24 h)	G1 vs. CG Zn↓, calcium↔, copper↔, selenium↔, iron↔, magnesium↔, viable cells↓, % dead cells↑, insulin↔ mRNA level of: cleaved PARP↑, BAX/Bcl-2↔, Mt1↔, Mt2↔, HO-1↓, Ogg1↔, p53↔, 8-OHdG↔, ɣ-H2AX↑, Ins1↔, Pdx1↔, Neurod1↔, Znt8↔
Wang et al., 2020 [57]	Cyprinus carpio	CG (*n* = 30)—arsenic trioxide 2.83 mg/L (orally, for 1 month) G1 (*n* = 30)—zinc chloride 1 mg/L and (orally, for 1 month) and arsenic trioxide 2.83 mg/L (orally, for 1 month)	G1 vs. CG kidney: BUN↓, creatinine↓, histopathological changes↓, DNA breaks↓, ROS↓, MDA↓, PC↓, 8-OHdG↓, SOD↑, GSH↑ mRNA and protein levels of: Bcl-2↑, Bax↓, Caspase 3↓, Caspase 8↓, Caspase 9↓, p53↓, PUMA↓, NRf2↓, GCL↓, NQO1↓, HO-1↓, iNOS↓, TNF-α↓, Il-10↓, Il-6↓, IĸB-α↑, nNF-ĸB↓, cNF-ĸB↑, p-ERK↔, p-p38↓, p-JNK1↔, p-JNK-2↔
Nasiry Zarrin Ghabaee et al., 2017 [58]	Rats, Wistar, adult, female	CG (*n* = 6)—sodium meta-arsenite 5 mg/kg bw/day (orally, for 42 days) G1 (*n* = 6)—zinc sulfate 20 mg/kg bw/day and (orally, for 42 days) and sodium meta-arsenite 5 mg/kg bw/day (orally, for 42 days)	G1 vs. CG infant total weight↑, infant tissue weight↑, total birth numbers↑, infant dead↓ kidney from pups: MDA↓, GSH↑, tubular damage↓, histopathological changes↓
Uthus and Nielsen 1985 [59]	Chicks, Golden Giant, cockerel	CG (*n* = 15)—zinc acetate 25 µg/g/diet (orally, for 28 days) and disodium arsenate 2 µg/g (orally, for 28 days) G1 (*n* = 15)—zinc acetate 2.5 µg/g/diet (orally, for 28 days) and disodium arsenate 2 µg/g (orally, for 28 days)	G1 vs. CG plasma: uric acid↑, urea↑ kidney: arginase↑
		CG (*n* = 15)—zinc acetate 25 µg/g/diet (orally, for 28 days) and disodium arsenate 2 µg/g (orally, for 28 days) G1 (*n* = 15)—zinc acetate 2.5 µg/g/diet for 9 day after that 10 µg/g for 19 days (orally) and disodium arsenate 2 µg/g (orally, for 28 days)	G1 vs. CG plasma: uric acid↑, urea↑ kidney: arginase↑
Zhao et al., 2019 [60]	Cyprinus carpio	CG (*n* = 30)—arsenic trioxide 2.83 mg/L (orally, for 1 month) G1 (*n* = 30)—zinc 1 mg/L and (orally, for 1 month) and arsenic trioxide 2.83 mg/L (orally, for 1 month)	G1 vs. CG heart: ROS↓, CAT↑, SOD↑, MDA↓, protein level of: Bax↓, BCl-2↑, Caspase 9↓, Caspase 3↓, LC3II/LC3I↓, p62↑, pI3K↑, p-AKT/AKT↑, p-mTOR/mTOR↑, p-38/p38↓, p-ERK/ERK↔, p-JNK/JNK↓ injury symptoms (agglutinated chromatin, damaged mitochondria and autophagosome)↓
Bhardwaj and Dhawan 2019 [61]	Rats, Wistar, adult, male	CG (*n* = 6)—sodium arsenite 100 mg/L in drinking water (orally, for 12 weeks) G1 (*n* = 6)—zinc sulfate in drinking water 227 mg/L (orally, for 12 weeks) and sodium arsenite 100 mg/L in drinking water (orally, for 12 weeks)	G1 vs. CG serum: Zn↑, Hb↔, reduced glutathione↑, CAT↑, MDA↓, GST↑, lymphocyte count↔, neutrophils↑, monocyte↔, eosinophilis↔, TLC↔ morphology of erythrocytes↑ morphological index of erythrocytes↓
Ahmad et al., 2013 [62]	Mice, Swiss-Webster, adult, female	CG (*n* = 10)—sodium arsenate 40 mg/kg bw/day in drinking water (orally, during pregnancy and until postnatal day 15) G1 (*n* = 10)—zinc sulfate 40 mg/kg bw/day in drinking water (orally, during pregnancy and until postnatal day 15) and sodium arsenate 40 mg/kg bw/day in drinking water (orally, during pregnancy and until postnatal day 15)	G1 vs. CG pups on postnatal days 21: bw↑, body hair appearance↓, eye opening↓, mean rotating reflex↓, mean righting reflex↓, mean cliff avoidance↓ male adolescent offspring (postnatal day 22): motor activity (number of squares crossed, wall rears, rears, movement duration)↑ serum: GGT↓, TBARS↓, GSH↑
Milton et al., 2004 [63]	2.3D cells (neuronal cell line)	CG—arsenic trioxide 20 µM G1—zinc sulfate 75 µM and arsenic trioxide 20 µM	G1 vs. CG DEVD-caspase activity↓
		CG—arsenic trioxide 20 µM G1—zinc sulfate 50 µM and arsenic trioxide 20 µM	G1 vs. CG DEVD-caspase activity↓
		CG—arsenic trioxide 20 µM G1—zinc sulfate 25 µM and arsenic trioxide 20 µM	G1 vs. CG DEVD-caspase activity↔
Fascineli et al., 2002 [64]	Mice, Swiss, adult, female	CG (*n* = 10)—sodium arsenate 45 mg/kg bw (IP, single dose on 8th gestation day) G1 (*n* = 10)—zinc sulfate 20 mg/kg bw (orally, on 7th and 8th gestation day) and after that sodium arsenate 45 mg/kg bw (IP, single dose on 8th gestation day) G2 (*n* = 10)—zinc sulfate 40 mg/kg bw (orally, on 7th and 8th gestation day) and after that sodium arsenate 45 mg/kg bw (IP, single dose on 8th gestation day)	G1 vs. CG maternal weight gain↓, fetal weight↔, signs of delayed ossification↔, placental weight↔, external, visceral and skeletal malformation↔, vertebrae skeletal anomalies↑
			G2 vs. CG maternal weight gain↔, fetal weight↔, signs of delayed ossification↔, placental weight↔, external, visceral and skeletal malformation↔, vertebrae skeletal anomalies↑
	Mice, Swiss, adult, female	CG (*n* = 10)—sodium arsenate 45 mg/kg bw (IP, single dose on 8th gestation day) G1 (*n* = 10)—zinc sulfate 5 mg/kg bw (orally, on 8th gestation day) and sodium arsenate 45 mg/kg bw (IP, single dose on 8th gestation day) G2 (*n* = 10)—zinc sulfate 10 mg/kg bw (orally, on 8th gestation day) and sodium arsenate 45 mg/kg bw (IP, single dose on 8th gestation day)	G1 vs. CG maternal weight gain↔, fetal weight↔, signs of delayed ossification↔, placental weight↔, external, visceral and skeletal malformation↔, vertebrae skeletal anomalies↑
			G2 vs. CG maternal weight gain↓, fetal weight↔, signs of delayed ossification↔, placental weight↓, external, visceral and skeletal malformation↔, vertebrae skeletal anomalies↑
	Mice CD-1, embryo culture	CG—sodium arsenite 5 µM G1—zinc chloride 500 µM and sodium arsenite 5 µM (6 h after zinc or simultaneously)	G1 vs. CG dysmorphology↔, lethality↔, neutral tube closure defects↔, pharyngeal arch dysmorphology↔, heart conotruncal dysmorphology↔
Beaver et al., 2017 [65]	Zebrafish, *Danio rerio,* embryos	CG—parental adults fish fed zinc 33.81 µg/g of diet (orally, for 8 weeks) and after that embryos was exposed to sodium arsenite 50 ppb (at 4 h to 120 h post fertilization) G1—parental adults fish fed zinc 14.45 µg/g of diet (orally, for 8 weeks) and after that embryos was exposed to sodium arsenite 50 ppb (at 4 h to 120 h post fertilization)	G1 vs. CG Zn↓, mRNA levels of: zip1↔, zip8↔, znt7↔ mortality↔, developmental malformation↔, activity of the embryos↓, at 24 h post fertilization: pax4↓ at 48 h post fertilization: mRNA levels of: nrf2a↔, nrf2b↓, Mt2↔, Ogg1↔, insa↔ at 120 h post fertilization: mRNA levels of: nrf2a↓, nrf2b↔, Mt2↔, Ogg1↔, insa↓, pdx1↔
		CG—parental adults fish fed zinc 33.81 µg/g of diet (orally, for 8 weeks) and after that embryos was exposed to sodium arsenite 500 ppb (at 4 h to 120 h post fertilization) G1—parental adults fish fed zinc 14.45 µg/g of diet (orally, for 8 weeks) and after that embryos was exposed to sodium arsenite 500 ppb (at 4 h to 120 h post fertilization)	G1 vs. CG Zn↓, mRNA levels of: zip1↔, zip8↔, znt7↔ mortality↔, developmental malformation↔, activity of the embryos↓ at 24 h post fertilization: pax4↔ at 48 h post fertilization: mRNA levels of: nrf2a↔, nrf2b↓, Mt2↔, Ogg1↓, insa↔ at 120 h post fertilization: mRNA levels of: nrf2a↔, nrf2b↔, Mt2↔, Ogg1↔, insa↔, pdx1↔
Nielsen et al., 1980 [66]	Chicks, day-old	CG—zinc acetate 40 µg/g/diet (orally, for 32 days) and sodium arsenate 2 µg/g (orally, for 32 days) G1—zinc acetate 5 µg/g/diet (orally, for 32 days) and sodium arsenate 2 µg/g (orally, for 32 days)	G1 vs. CG bw↓, liver wt/body wt ratio↔, hematocrit↑, growth↓ plasma: ALP↑
Altoe et al., 2016 [67]	Rats, Wistar, adult, male	CG (*n* = 6)—sodium arsenite 5 mg/kg bw/day (orally, for 60 days) G1 (*n* = 6)—zinc chloride 20 mg/kg bw/day (orally, for 60 days) and sodium arsenite 5 mg/kg bw (orally, for 60 days)	G1 vs. CG normal sperm morphology↑ abnormalities in spermatoza (wrong-angled hooks, folded sperm, amorphous head and normal tail, two heads)↓

↑—significant increase; ↓—significant decrease; ↔—no significant changes; ɣ-H2AX—gamma-H2A histone family member X; 8-OHdG—8-hydroxy-deoxyguanosine; Ache—acetylocholine; ACP—acid phosphatase; AKT—protein kinase B; ALAD—δ-aminolevulinic acid dehydratase; ALP—alkaline phosphatase; ALT—alanine transaminases; AST—aspartate transaminases; ATF6—activating transcription factor 6; ATG-5—autophagy related 5; Bax—Bcl-2-associated X protein; Bcl-2—B-cell lymphoma 2; BUN—blood urea nitrogen; bw—body weight; Ca—calcium; Ca^2+/^Mg^2+^-ATPase—calcium–magnesium adenosine triphosphatase; CAT—catalase; Ccl2—C-C motif chemokine ligand 2; CG—control group; CHOP—C/EBP homologous protein; cNF-ĸB—cytoplasmic nuclear factor kappa-light-chain-enhancer of activated B cells; Cu/Zn-SOD—copper zinc superoxide dismutase; CYP1A—cytochrome P-1A; Cyt—cytosols; DNA—deoxyribonucleic acid; eIF2α—eukaryotic initiation factor 2α; Fas—apoptosis antigen 1; G1—group 1; G2—group 2; GCL—glutamate cysteine ligase; GGT—ɣ-glutamyl transferase; GPx—glutathione peroxidase; GR—glutatione reductase; GRP78—glucose-regulating protein 78; GRP78—glucose-related protein 78; GRP94—glucose-related protein 94; GSH—glutathione; GSSG—oxidized glutathione; GST—glutathione s transferase; Hb—hemoglobin; HO-1—*heme oxygenase*-*1*; HSP60—heat shock response 60; HSP70—heat shock response 70; HSP90—heat shock response 90; ICAM1—intercellular adhesion molecule 1; IĸB-α—inhibitor of nuclear factor kappa B; Il-10—Interleukin 10; IL-1β—Interleukin 1β; IL-6—Interleukin 6; IL-8—Interleukin 8; iNOS—inducible nitric oxide synthase; IP—intraperitoneally; insa—insulin-a; Ins1—insulin-1; IRE1—inositol-requiring enzyme 1; LC3—microtubule-associated protein 1 light chain 3; MDA—malondialdehyde; Mic—microsomes; Mit—mitochondria; Mn-SOD—manganase superoxide dismutase; MT—metallothionein; Mt1—metallothionein 1; Mt2—metallothionein 2; mTOR—mammalian target of rapamycin; Na^+^/K^+^-ATPase—sodium-potassium adenosine triphosphatase; NADPH—nicotinamide adenine dinucleotide phosphate; Neurod1—neurogenic differentiation-1; NF-ĸB—nuclear factor kappa-B; nNF-ĸB—nuclear factor kappa-light-chain-enhancer of activated B cells; NQO1—NAD(P)H quinone dehydrogenase 1; nrf2—nuclearfactor(erythroid-derived2)-like 2; Nuc—nuclei; Ogg1—8-oxoguanine DNA-glycosylase 1; P62—sequestosome 1; PARP—poly(ADP) polymerase; pax4—paired box4; PC—protein carbonylation; pdx1—pancreatic and duodenal homeobox 1; p-eIF2a—Phospho-eukaryotic initiation factor 2 alpha; p-ERK—Phosphorylated Extracellular Signal-Regulated Kinase; PERK—PKR-like reticulum kinase; pI3K—phosphatidylinositol-3-kinase; p-JNK1—phosphorylated c-Jun N-terminal kinase 1; p-JNK-2 –phosphorylated c-Jun N-terminal kinase 2; p-PERK–Phospho- PKR-like ER kinase; PUMA—p53 upregulated modulator of apoptosis; ROS—reactive oxygen species; sc—subcutaneous; SOD—superoxide dismutase; tAs—total arsenic species; TBARS—thiobarbituric acid reactive substances; TLC—total leukocyte count; TNF-α—tumor necrosis factor-α; zip1 –zinc importer 1; zip8—zinc importer 7; Zn—zinc; ZnT—zinc transporter; znt7—zinc exporter 7; Znt8—zinc transporter 8; ZO-1—Zonula occludens-1; ZO-2—Zonula occludens-2; ZPP—zinc protoporphyrin.

## Data Availability

Not applicable.
